# Polyelectrolytes Are Effective Cryoprotectants for Extracellular Vesicles

**DOI:** 10.1021/acsami.4c11852

**Published:** 2024-12-12

**Authors:** Elżbieta Karnas, Mateusz Zając, Katarzyna Kmiotek-Wasylewska, Kamil Kamiński, Shin-Ichi Yusa, Sylwia Kędracka-Krok, Patrycja Dudek, Krzysztof Szczubiałka, Maria Nowakowska, Ewa K. Zuba-Surma

**Affiliations:** † Department of Cell Biology, Faculty of Biochemistry, Biophysics and Biotechnology, Jagiellonian University, 30-387 Krakow, Poland; ‡ Department of Physical Chemistry, Faculty of Chemistry, Jagiellonian University, 30-387 Krakow, Poland; § Department of Applied Chemistry, Graduate School of Engineering, University of Hyogo, Himeji, Hyogo 671-2280, Japan; ∥ Department of Physical Biochemistry, Faculty of Biochemistry, Biophysics and Biotechnology, Jagiellonian University, 30-387 Krakow, Poland

**Keywords:** extracellular vesicles, cryoprotection, polyelectrolytes, long-term storage, tissue regeneration

## Abstract

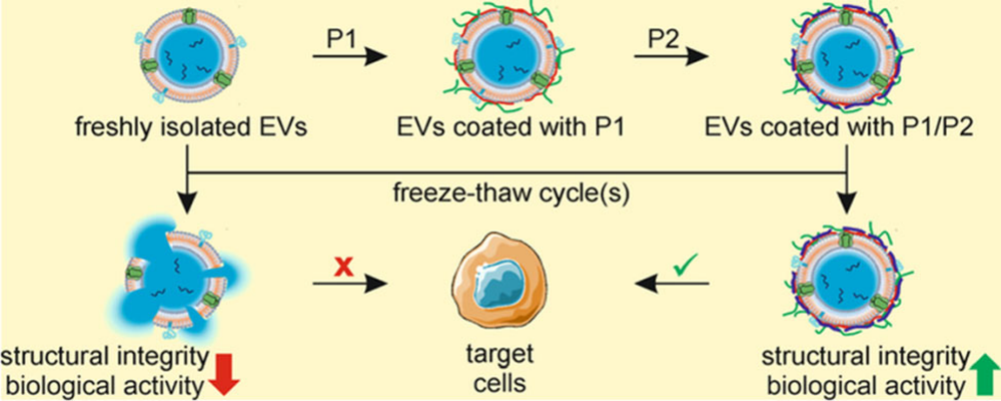

Extracellular vesicles (EVs) have been widely recognized as a heterogeneous group of membrane-coated submicrometer particles released by different types of cells, including stem cells (SCs). Due to their ability to harbor and transfer bioactive cargo into the recipient cells, EVs have been reported as important paracrine factors involved in the regulation of a variety of biological processes. Growing data demonstrate that EVs may serve as potential next-generation cell-free therapeutic factors. However, clinical application of EVs in tissue regeneration requires the development of standardized procedures for their long-term storage, without the loss of structural integrity and biological activity. In the current study, we developed a procedure of EV cryoprotection based on coating them with ultrathin polyelectrolyte bilayer consisting of cationic poly(ethylene glycol)-*block*- poly(3-(methacryloylamino)propyl)trimethylammonium chloride) (PEGn-*b*-PMAPTACm) and anionic of poly(2-acrylamido-2-methylpropanesulfonic acid) (PAMPS). Based on the nanoparticle tracking analysis, high-resolution flow cytometry, and mass spectrometry, we studied the vesicle integrity following single- or multiple freezing-thawing cycles and long-term storage. Additionally, we evaluated the effect of cryopreservation on the EVs functional activity in vitro. Obtained data indicate that coating with polyelectrolytes improves the structural integrity of EVs and preserves their biological activity in vitro. Additionally, proteomic analysis confirmed the effect of particle stabilization, as well as an enrichment in EV proteins in samples cryopreserved in the presence of tested polymers. Taking together, our study indicates that the application of polyelectrolytes may be a novel, effective way of facilitating long-term storage of EV preparations for their further use in the biomedical applications.

## 1 Introduction

Extracellular vesicles (EVs) are a heterogeneous group of alveolated structures that are released by different types of cells, including normal and malignant ones, as well as stem cells (SCs).^1^ EVs consist of a small volume of cytoplasm surrounded by a lipid bilayer with a composition similar to that of the cell membrane of their parental cells. Based on their size and the cellular compartment of their origin, EVs might be classified into a few groups, including apoptotic bodies (>1000 nm), microvesicles also called ectosomes (100–1000 nm), exosomes (30–150 nm) and other recently discovered small particles , such as migrasomes, exomeres, or exopheres.^2^ Such classification also results in differences in the biochemical composition of particular EV subsets. Nevertheless, the general arrangement and surface charge of biological membranes are similar in various types of mammalian cells, including human ones, consisting of a lipid bilayer containing mainly phospholipids and proteins with a balanced composition.^3,4^ Consequently, such a membrane similarity also exists in EVs secreted by those cells.

Importantly, an increasing number of reports indicates that EVs may harbor several types of bioactive factors, including lipids, proteins (e.g., transcription factors, enzymes, receptors, signaling and adhesive molecules), as well as nucleic acids (mRNA, miRNA, and other noncoding RNAs). Additionally, it has been widely demonstrated that such active cargo can be transferred by EVs to target cells, influencing their functional properties. Thus, EVs are considered important players in intracellular communication, being involved in the paracrine or autocrine regulation of a variety of biological processes. In that context, SC-derived EVs (SC-EVs) are of particular interest as a potential new-generation therapeutic alternative for whole cell-based therapies. It has been reported that SC-EVs may not only mimic the biological functions of their parental cells, including proregenerative capacity, but also possess several advantageous properties such as no tumorgenicity, lower immunogenicity, ability to cross blood-brain barrier, and can be delivered in the form of aerosols.^3−7^ Importantly, immunomodulatory and proregenerative capability of SC-EVs has been widely reported in several in vitro and preclinical in vivo models.^8−11^ As a consequence, there have been extensive attempts to introduce cell-free EV-based treatment strategies into a medical practice that would minimize the problems associated with immunogenicity, low retention, and possible adverse effects that may accompany whole cell transplantation. Additionally, it has been demonstrated that EVs may be used as protein and drug carriers^12−15^ that might be engineered in order to increase their effectiveness in the target site within the tissue.^16^ EVs are also considered as potential biosensors, biomarkers, and components of vaccines.^4−6,8,9^

Despite significant progress in the field, there are still several challenges associated with biomedical utilization of EVs. One of them is the need for standardized, effective methods of long-term storage conditions that would preserve structural integrity and biological activity of EV-based formulations.^17−20^ Several studies reported so far have demonstrated diverse influence of storage conditions on stability of EV samples, with results dependent on the type of biofluids as EV sources and their isolation procedure.^21^ However, current data do not provide clear indication on the effective method of EV cryoprotection that would preserve their structural and functional properties.

Thus, the aim of the current study was to provide the alternative approach for cryoprotection of EVs that is based on coating them with ultrathin layer/layers of selected, well-defined, and biocompatible polyelectrolytes. The cryoprotective effect of a single layer of poly(ethylene glycol-*block*-(3-(methacryloylamino)propyl)trimethylammonium chloride) block copolymer (PEG46-*b*-PMAPTAC52) adsorbed at the surface of EVs is presented. The enhancement of that effect accompanied by the improvement in the biocompatibility of such protected EVs was achieved by additional deposition of the second ultrathin anionic layer of poly(2-acrylamido-2-methylpropanesulfonic acid) (PAMPS18) using the layer-by-layer technique.^22,23^

## 2 Materials and Methods

Detailed experimental procedures are included in the Supporting Information.

### 2.1 Synthesis of the Polymers

PEG46-*b*-PMAPTAC52 [poly(ethylene glycol)-*b*-poly((3-(methacryloylamino)propyl)trimethylammonium chloride)] (referred to further as P1), its fluorescently labeled version, and PAMPS18 [poly(2-acrylamido-2-methylpropanesulfonic acid)] (referred to further as P2) were synthesized using a RAFT method (see Supporting Information for details; Figures S1 and S2).

### 2.2 Cell Culture

Human umbilical cord mesenchymal stem cells (hUC-MSCs) were isolated with explants method according to the previously described protocol^9^ and expanded in DMEM/F12 (Sigma-Aldrich, Saint Louis, MO, USA) medium with 10% of fetal bovine serum (FBS; Sigma-Aldrich). Primary human osteoblasts (HOBs; PromoCell, Heidelberg, Germany) were cultured in dedicated Osteoblast Growth Medium (OGM; PromoCell).

### 2.3 Isolation of EVs

EVs were isolated from the conditioned medium (CM) harvested from hUC-MSCs, using the sequential centrifugation method (Figure S3), as previously described.^9^ Obtained pellets containing EVs were resuspended in 0.22 μm filtered phosphate-buffered saline devoid of calcium and magnesium ions (PBS; Lonza, Basel, Switzerland). Prior to CM collection, hUC-MSCs were seeded and cultured in ultracentrifuged media in order to eliminate EVs and small particles of FBS origin. Media were ultracentrifuged (100,000*g*, 18 h, 4 °C), and the obtained media supernatants were then used for cell culture.

### 2.4 Coating of EVs with Polymers

Freshly isolated EVs were coated with one layer of the polymer, by mixing them at room temperature (RT) with P1 solution in PBS, to reach 50 μg/mL concentration of P1 and 10^11^ EVs/ml. For the two-layer coating, EVs previously coated with P1 were additionally coated with P2 in the same concentration (weight ratio of the P1 and P2 1:1). Sample containing EVs coated with a bilayer of P1 and P2 polymers is further referred to as P1/P2. EV suspension without polymers served as a control (Ctrl) sample.

### 2.5 Cryopreservation of EVs

Control EVs (Ctrl) or EVs coated with one (P1) or two (P1/P2) polymeric layers were stored at −80 °C for a minimum of 5 days. EV samples were frozen and thawed either one time (single freezing-thawing cycle) or multiple times (10 or 20×) prior to analyses. Additionally, a long-term storage (up to 8 months) approach was also performed, where samples were frozen and then thawed one time after 1, 2, 4, 6, and 8 months of storage in −80 °C.

### 2.6 Confocal Microscopy

Visualization of the EV coating with polymers was done with scanning confocal microscopy using cationic block copolymer labeled with Alexa Fluor 488 dye (PEG46-PMAPTAC52-AF488).

### 2.7 Nanoparticle Tracking Analysis (NTA)

The concentration and size distribution of EVs were measured with a NanoSight NS300 analyzer and NTA Software ver. 3.4 (Malvern Pananalytical, Malvern, UK), as previously described.^24^

### 2.8 High-Resolution Flow Cytometry

EVs were stained as previously described,^24^ with buffer containing SYTO RNASelect dye (Thermo Fisher Scientific) and one of allophycocyanin (APC)-conjugated mouse monoclonal antibodies: anti-anti-CD81 (clone 5A6), CD90 (clone 5E10), or appropriate isotype-match control (all from BioLegend, San Diego, CA, USA). Flow cytometry analysis was performed with an Apogee A60-Micro-PLUS cytometer and Histogram software (Apogee Flow Systems, Hemel Hempstead, UK).

### 2.9 Cytotoxicity Assay In Vitro

2 ×10^3^ of HOBs/well were treated with polymers for 24 h at the concentration of 50, 500, and 5000 μg/mL. Proliferation, cytotoxicity, and apoptosis of cells were assessed by ApoTox-Glo Triplex Assay kit (Promega, Madison, WI, US), according to the manufacturer’s protocol.

### 2.10 Viability Assay

2 ×10^3^ of HOBs/well were treated with freeze–thawed uncoated EVs (Ctrl) or EVs coated with P1 or P1/P2 in the dose of 1 × 10^9^ particles/well. Cells treated with freeze–thawed uncoated EVs (Ctrl fresh) served as a control. After 48 h, cell viability was measured using CaspGlo Luminescence Assay (Promega), according to the manufacturer’s protocol.

### 2.11 Metabolic Activity Assay

2 ×10^3^ of HOBs/well were treated with freeze–thawed uncoated EVs (Ctrl) or EVs coated with P1 or P1/P2 in the dose of 1 × 10^9^ particles/well. Cells treated with fresh uncoated EVs (Ctrl fresh) served as a control. After 4 h, the ATP concentration in cells was measured by the ATPLite Luminescence Assay System (PerkinElmer), according to the manufacturer’s protocol.

### 2.12 Cytoprotection Assay

2 ×10^3^ of HOBs/well were treated with staurosporine as an inducer of apoptosis (1 μM; Sigma-Aldrich) for 4 h. Next, freeze–thawed uncoated EVs (Ctrl) or EVs coated with P1 or P1/P2 in the dose of 1 × 10^9^ particles/well. Cells treated with fresh uncoated EVs (Ctrl fresh) served as a control. After 4 h, cell viability was measured using CaspGlo Luminescence Assay (Promega), according to the manufacturer’s protocol.

### 2.13 Proteomic Analysis

Proteome of purified EV control (uncoated) or P1/P2-coated samples subjected to a single freezing-thawing cycle and fresh uncoated EVs (Ctrl fresh) were analyzed using LC–MS/MS. Following freezing-thawing, EV samples were ultracentrifuged (100,000*g*, 70 min at 4 °C) to separate pellets containing intact EVs, from supernatants containing proteins released by the potential disruption of EVs. Detailed procedure is described in the Supplementary Methods.

### 2.14 Statistical Analysis

Unless otherwise stated, all experiments were repeated three times. The exact number of repetitions (*N*) is indicated in the figure captions. Data on the bar graphs present a mean ± standard deviation (SD). Graphs were performed using GraphPad Prism 5 Software (GraphPad Software Inc., San Diego, CA, USA). Statistical significance was calculated by Statistica Software (StatSoft, Tulsa, OK, USA) using a two-tailed Student’s *t* test or a *t* test with a fixed reference value of 100% (for the data expressed as percentage of control). The value of *p* < 0.05 was considered significant and expressed on figures as black asterisks.

## 3 Results

### 3.1 Coating of EVs with Ultrathin Polyelectrolyte Layers

**Figure 1:**
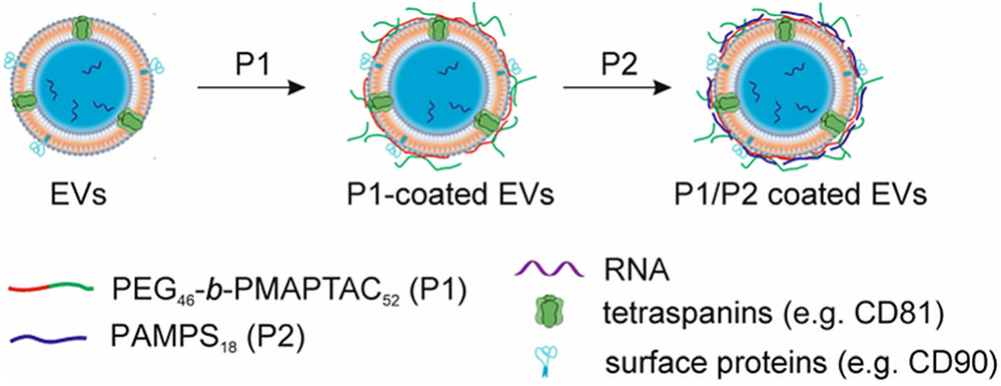
Schematic representation of EV coating with the ultrathin P1 layer or P1/P2 bilayer of the designed polymers.

Well-defined fraction of EVs secreted by hUC-MSCs was obtained from CM by sequential centrifugation (Figure S3). The mean diameter of freshly isolated EVs assessed using NTA was 150.2 ± 6.7 nm (Figure S4). Freshly isolated EVs were coated with single or double polyelectrolyte layers (Figure 1). The EVs were first covered with a PEG_46_-*b*-PMAPTAC_52_ layer (P1), which was deposited on their surface and stabilized mainly by the attractive electrostatic interactions between negatively charged surfaces of EVs and positively charged polymers. To increase the stability of such protected vesicles and to improve their biocompatibility and stability of their aqueous dispersion, EVs were additionally covered with polyanionic PAMPS_18_ (P2), together forming a P1/P2 bilayer coating. These polyelectrolytes were selected based on the preliminary screening experiments carried out with the use of a series of polymers varying in structure and composition.

The formation of a polymeric coating on the EVs was visualized by using laser scanning confocal microscopy imaging. Upon addition of Alexa Fluor 488-labeled P1 to the EV suspension, the presence of fluorescent particles was observed (Figure S5A), confirming coating of EVs with the polymer, while no signal for uncoated EVs (Figure S5B) and for P1 solution was observed (Figure S5C). The coating procedure was followed by measuring the changes in the zeta potential of EVs after both coating steps. The zeta potential of freshly isolated EVs amounted at −17.3 ± 0.3 mV. The zeta potential of EVs coated with a single P1 layer increased (became less negative) to −8.2 ± 0.4 mV, while that for EVs coated with the P1/P2 bilayer decreased (became more negative) to −13.7 ± 0.9 mV (Table S1). Such changes of zeta potential confirm the coating of EVs with the charged polymers. The repulsive electrostatic interactions and steric hindrance of polyelectrolyte modified EVs are key factors in stabilization of a vesicle aqueous dispersion.

### 3.2 Influence of Polymers on the Structural Integrity and Phenotype of EVs Subjected to Single Freezing-Thawing Process

**Figure 2:**
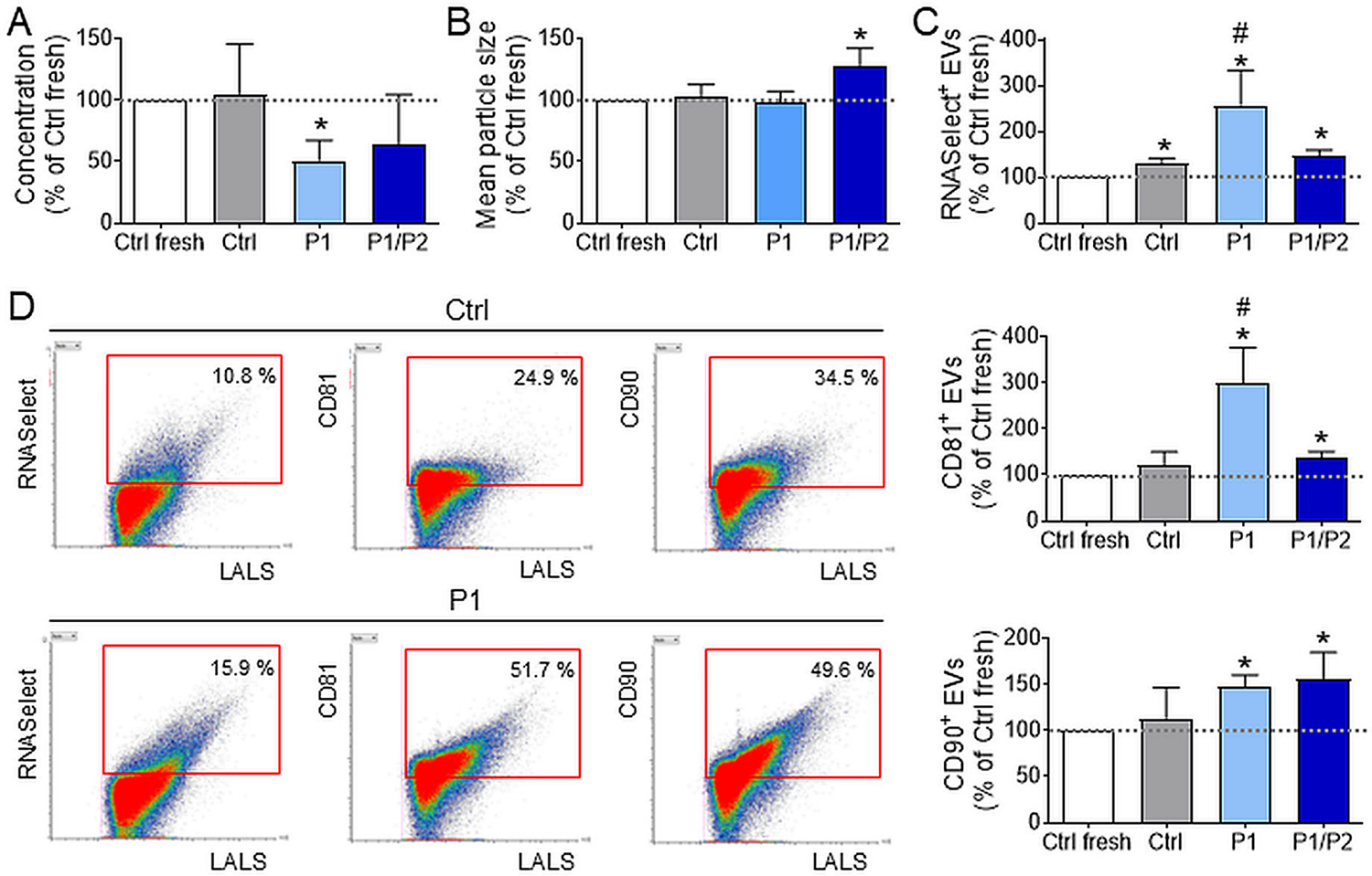
Effect of a single freezing-thawing cycle on EVs in the presence of polymeric cryoprotectants P1 and P1/P2. NTA analysis of particle concentration (A) and mean size (B) in EV samples after freezing-thawing process. Data (mean ± SD; *N* = 3) are presented as % relative to Ctrl fresh sample (uncoated EVs, no freezing), indicated by the gray dotted line (100%). (C) Flow cytometry analysis of the influence of cryoprotectants on the integrity of EVs, assessed by RNASelect dye staining, as well as the presence of antigens typical for EVs (CD81) and mesenchymal cells (CD90). Data (mean ± SD; *N* = 3) are presented as % relative to Ctrl fresh sample (uncoated EVs, no freezing), indicated by the gray dotted line (100%). (D) Representative dot plots for Ctrl sample (no polymer) and EVs coated with P1 polymer, after one freezing-thawing cycle. Percentage of objects positive for the analyzed markers is shown in the red gates. LALS - low angle light scattering signal, proportional to the relative particle size. *P < 0.05 vs Ctrl fresh. ^#^*P* < 0.05 vs Ctrl (uncoated) EVs.

In order to investigate the effect of coating with polymers on structural stability of EVs following their cryopreservation, EVs coated with either a single P1 layer or a double polymer layer (P1/P2) were first subjected to a single freezing-thawing cycle. NTA analysis has demonstrated a decrease in particle concentration in EVs coated with P1 (*p* < 0.05) and P1/P2 (*p* > 0.05) following freezing-thawing procedure (Figure 2A). Additionally, particle size was significantly elevated in P1/P2 samples (128.3 ± 13.9% of Ctrl fresh; 193.7 ± 33.5 nm; Figures 2B and S6A), which may be a consequence of the presence of polymer double layer together with accompanying hydrating water and counterions that surround EV particles, increasing their hydrodynamic diameter.^25^ This conclusion is supported by the fact that an increase in the mean size of particles (hydrodynamic diameter) was also observed following coating of freshly isolated EV samples (Figure S6B).

Next, an influence of the polymer coating on the integrity and phenotypic characteristics of cryopreserved EV preparations was also investigated using high-resolution flow cytometry. To do so, EVs were stained with RNASelect dye, which, after penetration into undamaged EVs, selectively binds to RNA molecules, exhibiting green fluorescence.

The obtained results show an increase in the percentage of RNASelect-positive objects following a single freezing-thawing cycle, both for uncoated EVs as well as for EVs coated with P1 and P1/P2, when compared to the freshly isolated EVs (Figure 2C, upper panel). Importantly, the percentage of RNA Select+ particles (intact EVs) was the highest for P1-coated EVs and significantly higher (2-fold) than for uncoated sample (Ctrl). Similarly, the percentage of RNA Select+ objects was slightly elevated for EVs coated with P1/P2, when compared to Ctrl sample (*P* > 0.05). Considering concomitant decrease in the particle concentration following freezing-thawing cycle of EVs coated with polymers, increase in the RNASelect-positive objects may be an effect of polymer-based promotion of nonvesicular objects disintegration (including contaminants such as protein aggregates) that are not stained with RNASelect.^26−28^ Thus, degradation of these contaminating objects would result in an observed increase in the percentage of RNASelect+ vesicles in the sample following freezing-thawing procedure.

Additionally, we have also evaluated the effect of EV cryopreservation in the presence of tested polymers on the selected phenotypic properties of EVs. The presence of selected surface antigens was determined by subjecting EVs to the immunofluorescence staining with appropriate antibodies, such as those directed against CD81 as this tetraspanin is considered to be an EV marker, as well as against CD90 which is an antigen typical for mesenchymal cells, that are parental cells secreting EVs used in this study. First, we did not observe any effect of polymers on the binding of antibodies into CD81 and CD90 antigens in freshly isolated EV samples (Figure S7). However, there was a significantly higher percentage of both CD81-positive and CD90-positive objects after single freezing-thawing of EVs covered with P1 and P1/P2, comparing to the freshly isolated EVs, indicating protective effect of polymers (Figure 2C, middle and lower panels; Figure 2D). Additionally, the percentage of CD81-positive EVs was significantly higher in the samples cryopreserved following coating with P1, when compared to the frozen uncoated ones (Figure 2C, middle panel). Taking together, the obtained results suggest that the P1 layer and P1/P2 bilayer stabilize EVs and enhance their cryopreservation during single thawing procedure.

### 3.3 In Vitro Cytotoxicity Analysis of Polymers

**Figure 3:**
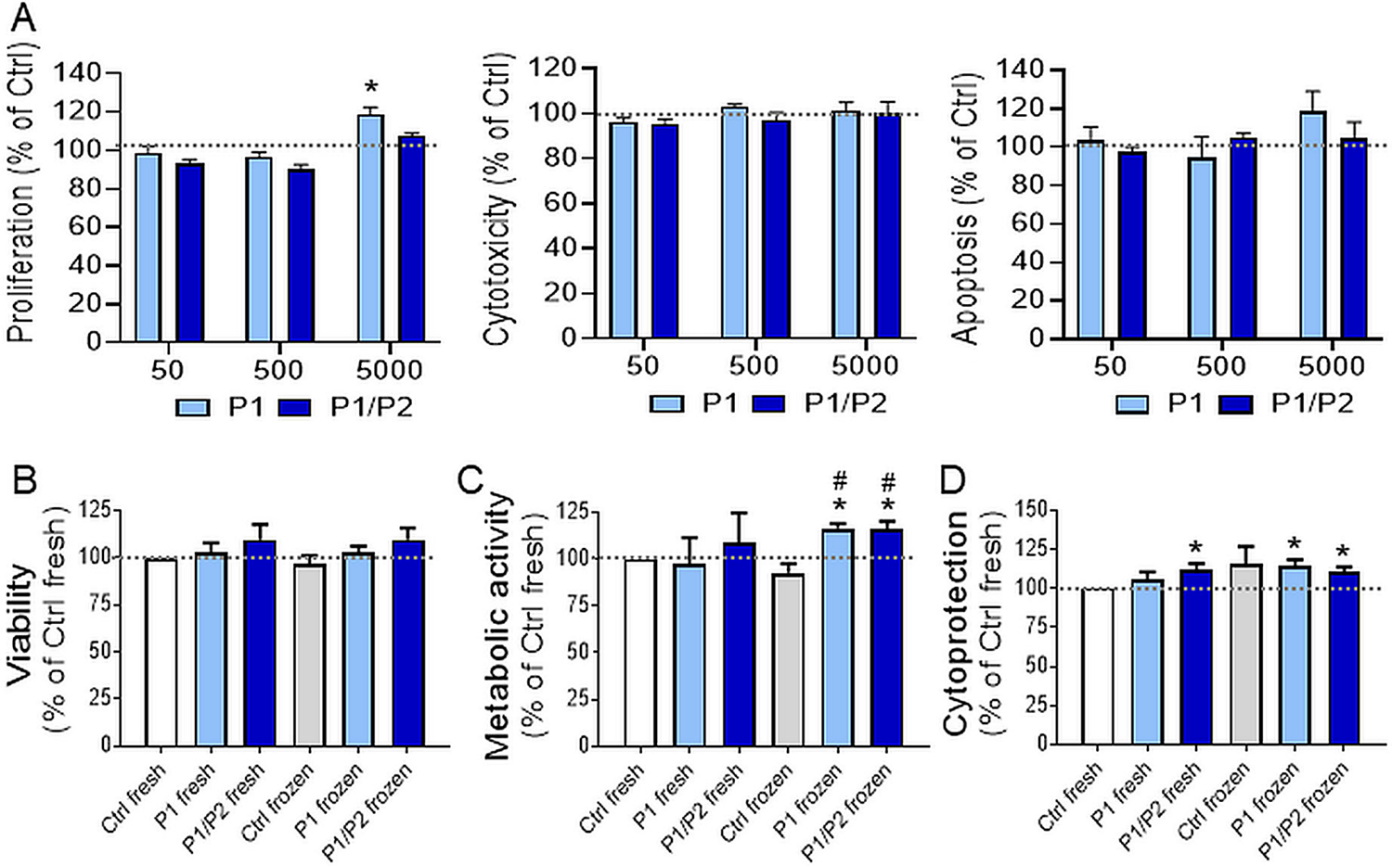
Influence of P1 and P1/P2 polymers on selected functional properties of human osteoblasts (HOBs) in vitro. (A) Effect of P1 and P1/P2 on HOBs proliferation, cytotoxicity, and apoptosis, analyzed by ApoTox-Glo Triplex Assay kit 24 h post addition of polymers into the culture medium at the concentration of 50, 500, and 5000 μg/mL. Data (mean ± SD; *N* = 3) are presented as % relative to Ctrl (no polymer), indicated by the gray dotted line (100%). (B–D) Influence of polymer-based cryopreservation on the functional activity of EVs. Freshly isolated or single frozen–thawed EVs were added to the HOBs culture at a dose of 1 × 10^9^ EVs per 2 × 10^3^ cells. (B) Analysis of HOBs viability after 48 h of incubation with EVs, measured using the Caspase-Glo 3/7 kit. (C) Influence of EVs on the metabolic activity of HOBs, measured by the ATPLite kit 4 h after the addition of EVs. (D) Cytoprotective effect of EVs on HOBs. 4 h before the addition of EVs, cells were treated with staurosporine as an inducer of apoptosis. 4 h after the addition of EVs, cell viability was measured using the Caspase-Glo 3/7 kit. Data in the graphs (mean ± SD; *N* = 3) are presented as % relative to Ctrl fresh sample (uncoated EVs, no freezing), indicated by the gray dotted line (100%). *P < 0.05 vs Ctrl fresh. ^#^*P* < 0.05 vs Ctrl frozen EVs.

Considering the possible therapeutic application of polymer-coated EVs and our current scientific interest in the development of EV-based treatment of bone defects and osteoarthritis, in the next step, we have investigated the potential cytotoxicity of tested polymers on human osteoblasts (HOBs) as target cells. We performed an assay in which HOBs were incubated with different concentrations of P1 or P1/P2 for 24 h. After that time, proliferation, cytotoxicity, and level of apoptosis were assessed. We observed that both P1 and P1/P2 at the concentrations corresponding to 1×, 10×, and 100× of the dose used for EVs coating (50, 500, and 5000 μg/mL, respectively) showed no cytotoxic and no proapoptotic influence on the tested cells (Figure 3A). Interestingly, the addition of P1/P2 polymer to the culture medium in the concentration of 5000 μg/mL caused an elevated level of cell proliferation when compared to the untreated cells. These data demonstrate the safety of tested polymers in the context of their use for the coating of EV preparations for the purpose of further functional studies.

### 3.4 Analysis of the Influence of Polymers on the Functional Properties of EVs

Cryopreservation is one of the key methods in preserving stability of EV formulations for their subsequent use, both in basic research and in biomedical applications. Many experimental systems have demonstrated the effect of EVs secreted by stem cells on selected functional properties of target cells. Particularly, an influence of EVs secreted by hUC-MSCs on the several biological processes in the recipient cells has been widely reported.^29,30^ Therefore, we analyzed the influence of tested polymers on the cryopreservation efficiency of EVs from hUC-MSCs, in the context of their biological efficacy in vitro. For that purpose, we investigated functional effectiveness of these EVs, using human osteoblasts (HOBs) as exemplary target mammalian cells. EVs coated with P1 or P1/P2 layers as well as the control ones (uncoated) were cryopreserved by single freezing-thawing cycle and then added to the HOB cell culture. Their influence on the selected properties of HOBs was then compared with the freshly isolated EVs. Obtained data indicate slightly improved viability of cells treated with freshly isolated and cryopreserved EVs coated with P1/P2 (*p* > 0.05; Figure 3B). On the other hand, cryopreserved EVs coated with both P1 and P1/P2 improved metabolic activity of HOBs significantly better, when compared to the uncoated EVs (Figure 3C). Similarly, coating EVs with tested polymers enhanced their cytoprotective effect on HOBs treated with apoptosis inducer (Figure 3D). Taking together, we demonstrated that the coating with P1 and P1/P2 has a positive effect on the functional properties of hUC-MSC-derived EVs subjected to single freezing-thawing cryopreservation in vitro.

### 3.5 Proteomic Analysis

**Figure 4:**
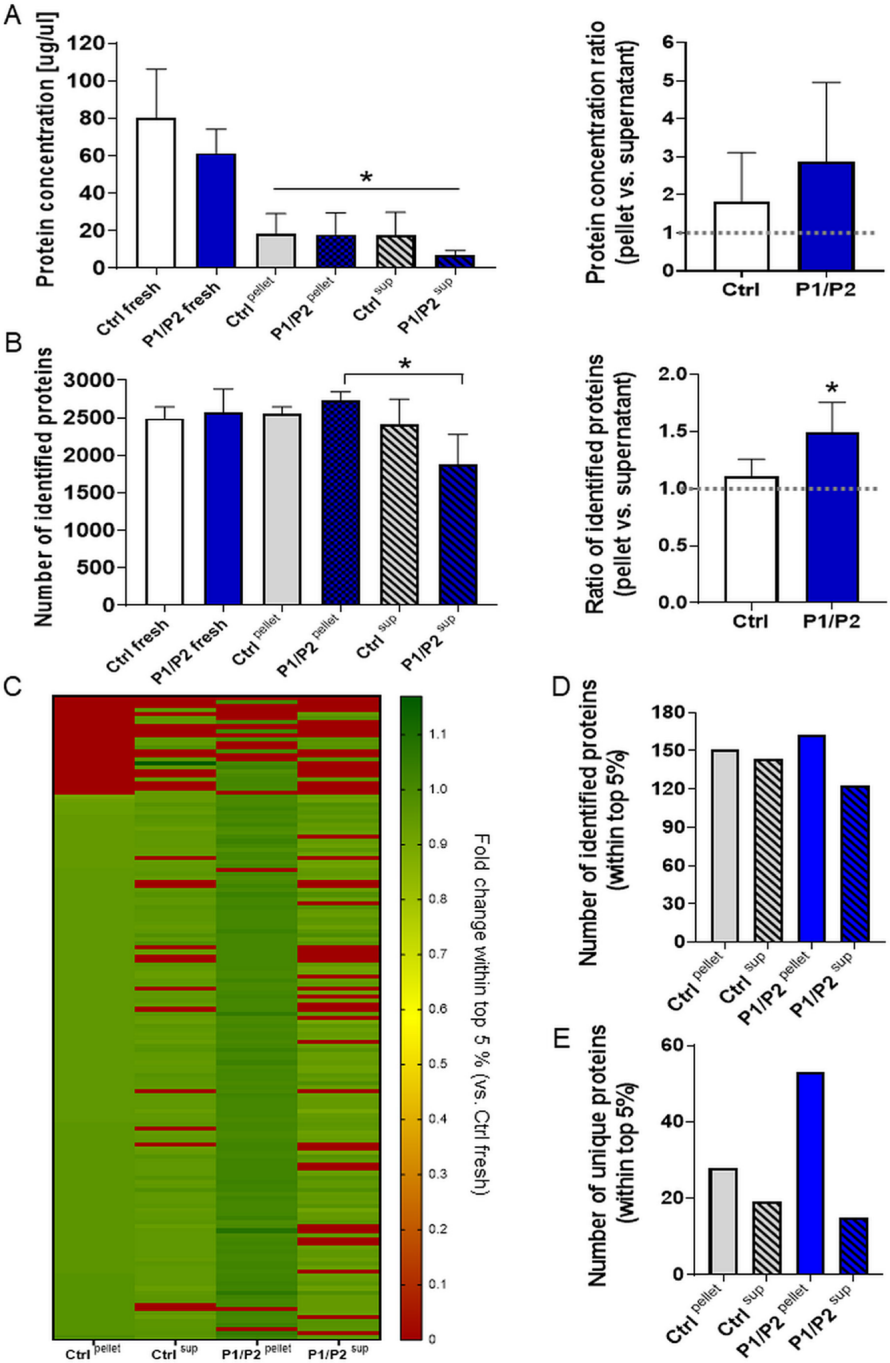
Proteomic analysis of cryopreserved EVs coated with P1/P2. Purified EVs were coated with a P1/P2 bilayer followed by a single freezing-thawing cycle. After thawing, samples were ultracentrifuged to separate EV pellet (containing undamaged vesicular fraction) from supernatants (containing proteins released following vesicle rupture). (A) Comparison of total protein concentration in the analyzed samples (left panel) and the ratio of protein concentration in pellets and in supernatants (right panel). Data in the graphs are presented as mean ± SD (*N* = 3). *P < 0.05 vs Ctrl fresh. (B) Comparison of the numbers of identified proteins in the analyzed samples (left panel) and the ratio of the numbers of identified proteins in pellets and in supernatants (right panel). Data in the graphs are presented as mean ± SD (*N* = 3). *P < 0.05 for pellet vs supernatant. (C) Heatmap analysis comparing abundance of top 5% proteins (selected based on the top iBAQs) samples, including proteins that are both common and unique for particular sample types. Fold change of mean iBAQ values (*N* = 3) was calculated relative to Ctrl fresh sample (uncoated EVs, no freezing; fold change = 1). Fold change of 0 indicates that particular proteins were undetectable within top 5% of proteins. (D) Comparison of the number of identified proteins within the top 5% proteins (selected based on the top iBAQs). (E) Comparison of the number of proteins within the top 5% proteins (selected based on the top iBAQs) that are uniquely identified either in pellet or supernatant.

As the freezing-thawing procedure may have a negative impact on the structural stability of EVs, causing their disruption due to the formation of water crystals and consequently the qualitative and quantitative changes in their protein cargo, we employed proteomic MS analysis to further investigate the effect of EV coating with tested polymers on the preservation of their integrity following single freezing-thawing cycle after storage in −80 °C. Coating of EV samples with P1/P2 was selected due to the more negative zeta potential, which indicates higher stability than that of P1-coated vesicles (zeta potential of −13.7 ± 0.9 mV vs −8.2 ± 0.4 mV, respectively; Table S1). Importantly, the zeta potential of EVs coated with P1/P2 was closer to that of the native EVs (−17.3 ± 0.3 mV, Table S1). Freshly isolated EVs were divided into an uncoated control sample and a sample coated with P1/P2. Additionally, prior to coating and freezing, EVs were purified by qEV columns from potential protein coisolates to eliminate protein background (mainly albumins) that may interfere with proteomic analysis. Next, following a single freezing-thawing cycle, EV samples were subjected to ultracentrifugation which allowed separation of the samples into pellets and supernatants. We assumed that pellets contained intact EVs, whereas EVs that underwent disruption following freezing and thawing released their protein cargo into the supernatant.^19^ First, we compared the concentration of total proteins in samples obtained from the same quantity of EVs. As expected, the concentration of proteins in thawed samples that were split into pellets and supernatants was lower than in the freshly isolated samples, which indicates a partial disruption of vesicular fraction following freezing-thawing cycle (Figure 4A left panel).

Importantly, the ratio of protein concentration in pellets and in supernatants was higher for EVs cryopreserved following coating with P1/P2 bilayer, when compared to the frozen uncoated samples (Figure 4A right panel; *p* > 0.05). We have also analyzed the number of proteins identified in the subsequent samples, demonstrating that the number of proteins present in the supernatant of P1/P2-coated EVs (P1/P2^sup^) was significantly lower than in the pellet (P1/P2 ^pellet^), while the number of identified proteins was comparable between the pellet (Ctrl ^pellet^) and supernatant of uncoated EVs (Ctrl ^sup^; Figure 4B left panel). Analogically, the ratio between the number of identified proteins in pellets and supernatants was significantly higher for EVs cryopreserved following coating with the P1/P2 bilayer, when compared to the frozen uncoated EV samples (Figure 4B; right panel), which indicates a protective effect of P1/P2 on the EV integrity. Importantly, comparison of proteins present in pellets indicated that 2137 proteins were common for both Ctrl and P1/P2-coated EVs. However, the number of proteins unique only for P1/P2 ^pellet^ (absent in Ctrl ^pellet^, 634 proteins) was 6.4 times higher than the number of proteins present only in Ctrl ^pellet^ (99 proteins; Figure S8 left panel).

On the contrary, the analysis performed for supernatants revealed that there were 1687 common proteins, but the number of proteins unique only for P1/P2 ^sup^ (absent in Ctrl ^sup^, 188 proteins) was 1.9 times lower than the number of proteins present only in Ctrl ^pellet^ (348 proteins; Figure S8 right panel). These data confirm our observation on the enrichment of the number of proteins in pellets (assumed as containing intact vesicles) following EVs cryopreservation with the P1/P2 bilayer.

Furthermore, the detailed analyses were performed on the narrowed pool of 5% the most abundant proteins present in the particular samples after their freezing-thawing and separation into pellet and supernatant fractions. Direct comparison of protein abundance indicated their differential distribution between pellets and supernatants with relatively the highest abundance in P1/P2 ^pellet^ and the lowest in P1/P2 ^sup^ (Figure 4C). Analogically, the highest number of top 5% proteins was identified for P1/P2 ^pellet^ and the lowest for P1/P2 ^sup^ (Figure 4D). Interestingly, the highest disproportion between number of unique proteins (identified within top 5% either in only pellet or only supernatant) was also observed for EVs coated with P1/P2 (53 proteins unique for P1/P2 ^pellet^ and 18 unique for P1/P2 ^sup^), whereas this effect was much lower for uncoated EVs (28 proteins unique for Ctrl ^pellet^ and 19 unique for Ctrl ^sup^; Figure 4E).

**Figure 5:**
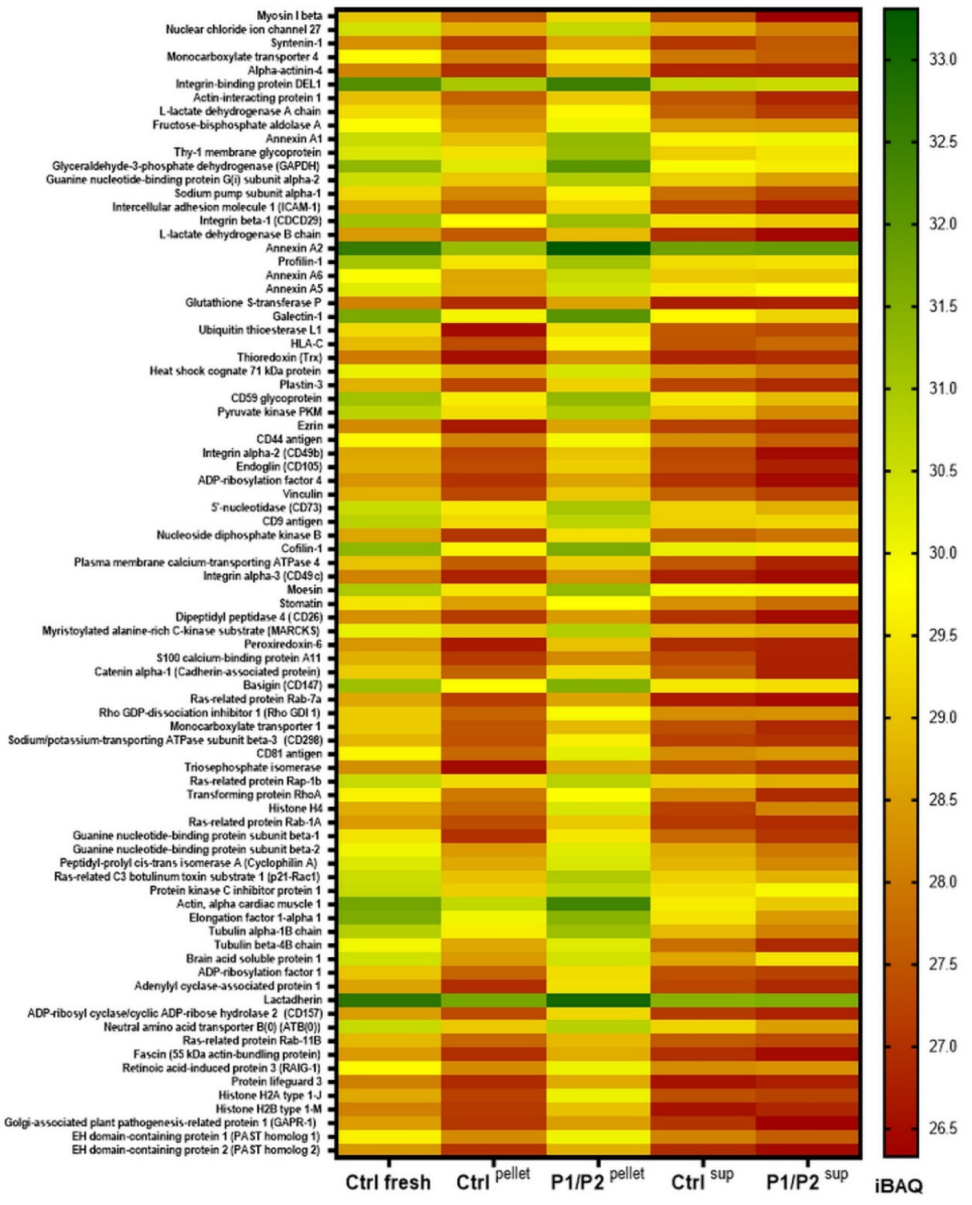
Comparison of the abundances of proteins present in all sample types. Heatmap analysis comparing mean iBAQ values (*N* = 3) for the top 5% proteins (selected based on the top iBAQs) between samples.

Subsequently, we evaluated the abundance of proteins that were common for all analyzed samples within the top 5%, including Ctrl fresh sample. Again, we observed the similar pattern of protein abundance between Ctrl fresh and P1/P2 ^pellet^ (Figure 5).

Protein abundance in those samples was also higher than that in Ctrl ^pellet^ and Ctrl ^sup^ samples. Moreover, the lowest abundance among common proteins was observed for P1/P2 ^sup^ (Figure 5), which supports the concept of the protective role of the polymer bilayer (P1/P2), which reduces EV disruption and protein release following the freezing and thawing procedure.

**Figure 6:**
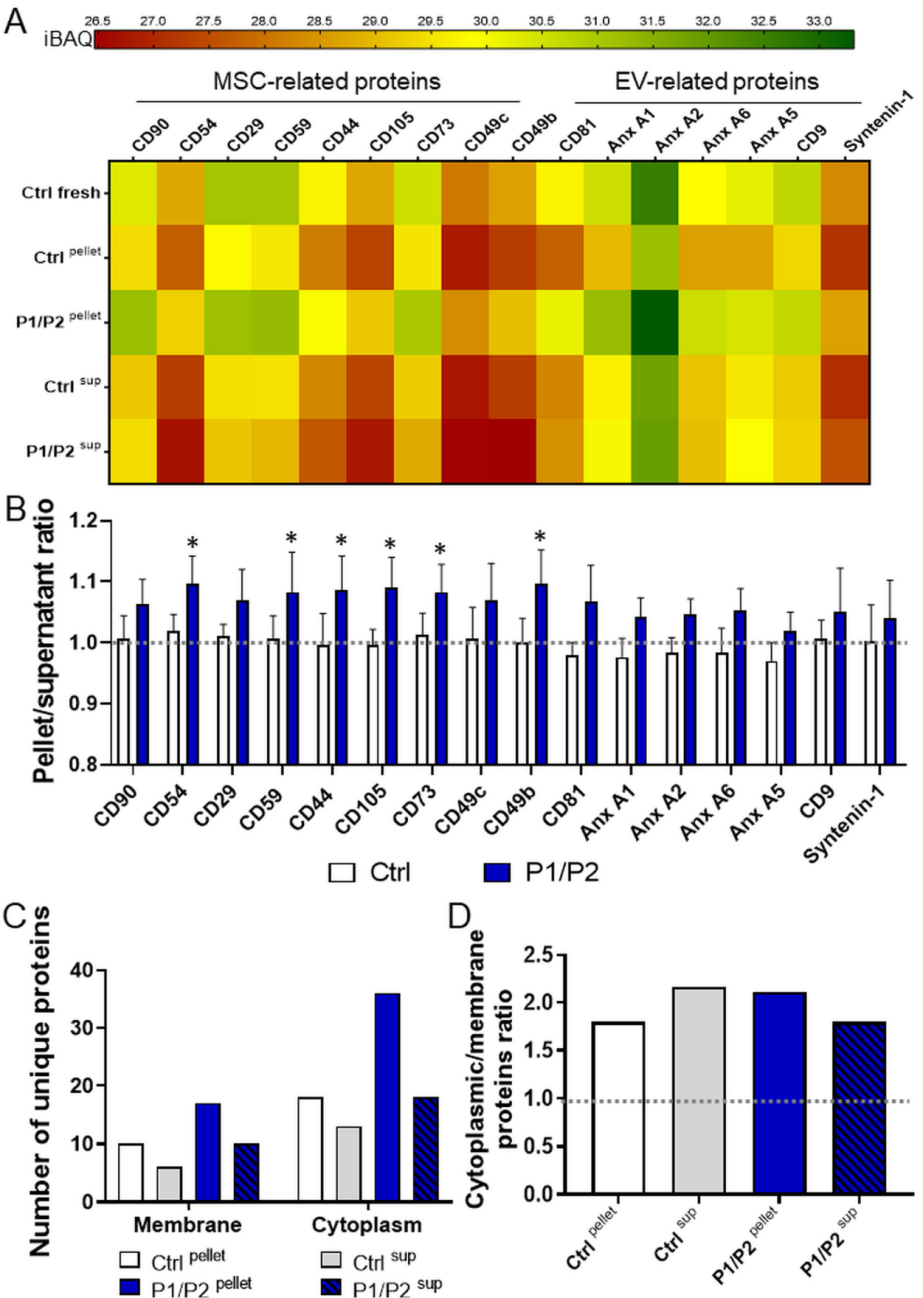
Qualitative analysis of the protein distribution. (A,B) Distribution of markers of EVs and their parental MSCs common for all samples within top 5% of identified proteins. (A) Heatmap analysis comparing mean iBAQ values (*N* = 3) for the selected proteins between samples. (B) Comparison of pellet/supernatant iBAQ ratio of selected proteins. (C,D) Comparison of abundance of membrane and cytoplasmic proteins uniquely identified in either pellet or supernatant. (C) Number of unique cytoplasmic and membrane proteins in particular samples. (D) Ratio of number of identified cytoplasmic and membrane proteins. *P < 0.05 for pellet vs supernatant.

Additionally, we also performed qualitative analysis of protein content within 5% the most abundant, identifying 9 proteins known as MSC markers, such as CD29, CD73, CD90, CD105, and others.^31^ Additionally, we found 7 proteins related to the EV markers, including tetraspanins (CD9, CD81), annexins, and syntenin.^32^ Importantly, the comparison of protein abundance revealed the enrichment of MSC- and EV-related proteins in P1/P2 ^pellet^, when compared to P1/P2 ^sup^, while such enrichment in pellet was not visible for uncoated EV samples (Figure 6A,B). Interestingly, among proteins that were uniquely identified either in pellet or in supernatant within 5% the most abundant proteins, there was a higher number of cytoplasmic proteins than membrane ones in both uncoated and P1/P2-coated samples (Figure 6C,D).

Thus, proteomic analyses have confirmed that coating of EVs with a P1/P2 bilayer stabilizes their structure during a single freezing-thawing cycle, reducing the loss of protein cargo following EV disruption. It also quantitatively and qualitatively changes the protein content of EVs.

### 3.6 Analysis of the Cryoprotective Effect of Polymers on the Stability of EV Samples Subjected to Multiple Freezing-Thawing Cycles

**Figure 7:**
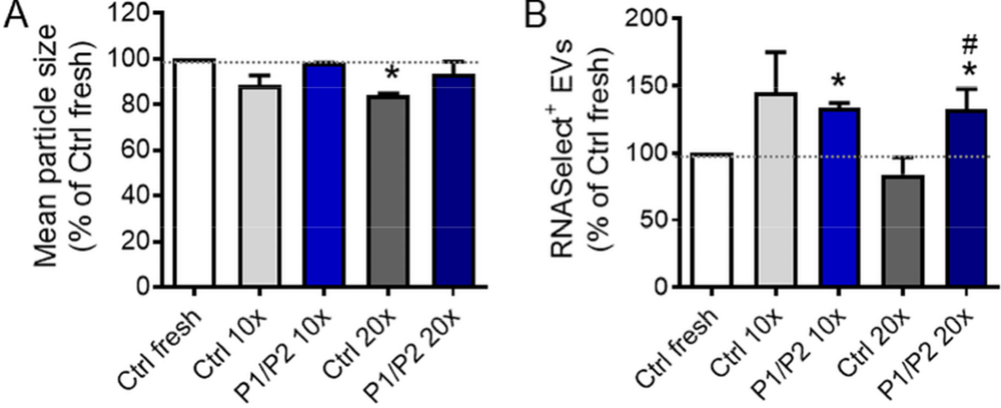
Effect of multiple freezing-thawing of EVs in the presence of P1/P2. Prior analysis, EVs were coated with P1/P2 and followed 10 or 20 freezing-thawing cycles. (A) NTA analysis of mean particle size. (B) Flow cytometry analysis of the EV integrity, assessed by RNASelect dye staining. Data on graphs (mean ± SD; *N* = 3) are presented as % relative to Ctrl fresh sample (uncoated EVs, no freezing), indicated by the gray dotted line (100%). *P < 0.05 versus Ctrl fresh. ^#^*P* < 0.05 vs Ctrl (uncoated) EVs.

Apart from the cryoprotective effect of P1/P2 on EVs subjected to a single freezing-thawing cycle, we investigated the effect of coating EVs with P1/P2 on their integrity following multiple freezing-thawing cycles. This is of particular importance regarding the potential necessity to frequently defrost the same EV sample in laboratory practice. Multiple freezing-thawing cycles may also be considered as a kind of a “stress test” potentiating the effects of a single cycle. Freshly isolated EV samples were mixed with polymers and then subjected to 10 or 20 freezing-thawing cycles. NTA analyses have revealed that the average size of particles was slightly lower for control (uncoated) samples following 10 freezing-thawing cycles, with the significant size reduction after 20 freezing-thawing cycles, which may indicate partial disintegration of vesicles (Figure 7A). On the other hand, there was no substantial change in the mean size of particles in EV samples coated with P1/P2, after both 10 and 20 cycles of freezing-thawing, when compared to the unfrozen sample (Figure 7A).

Additionally, flow cytometry analysis demonstrated a significantly higher percentage of RNASelect-positive particles in EV samples coated with P1/P2 and subjected to 10 or 20 freezing-thawing cycles, when compared to the unfrozen EV sample, whereas such a significant effect was not observed for uncoated EVs (Figure 7B). Moreover, the percentage of RNASelect-positive particles was also significantly higher in P1/P2-coated samples subjected into 20 cycles freezing-thawing, comparing to the analogical uncoated EVs. These data indicate considerable protective effect of P1/P2 on multiply frozen–thawed EVs.

### 3.7 Analysis of the Influence of Polymers on the Structural Integrity and Phenotype of EVs Subjected to Long-Term Storage

Long-term storage of EV preparations carries a risk of a gradual loss of their integrity and biological activity. Thus, in order to evaluate the effect of P1/P2 on the biological properties of EVs subjected to long-term cryopreservation, we analyzed selected properties of EV preparations stored at −80 °C for up to 8 months, thawing them in subsequent intervals.

**Figure 8:**
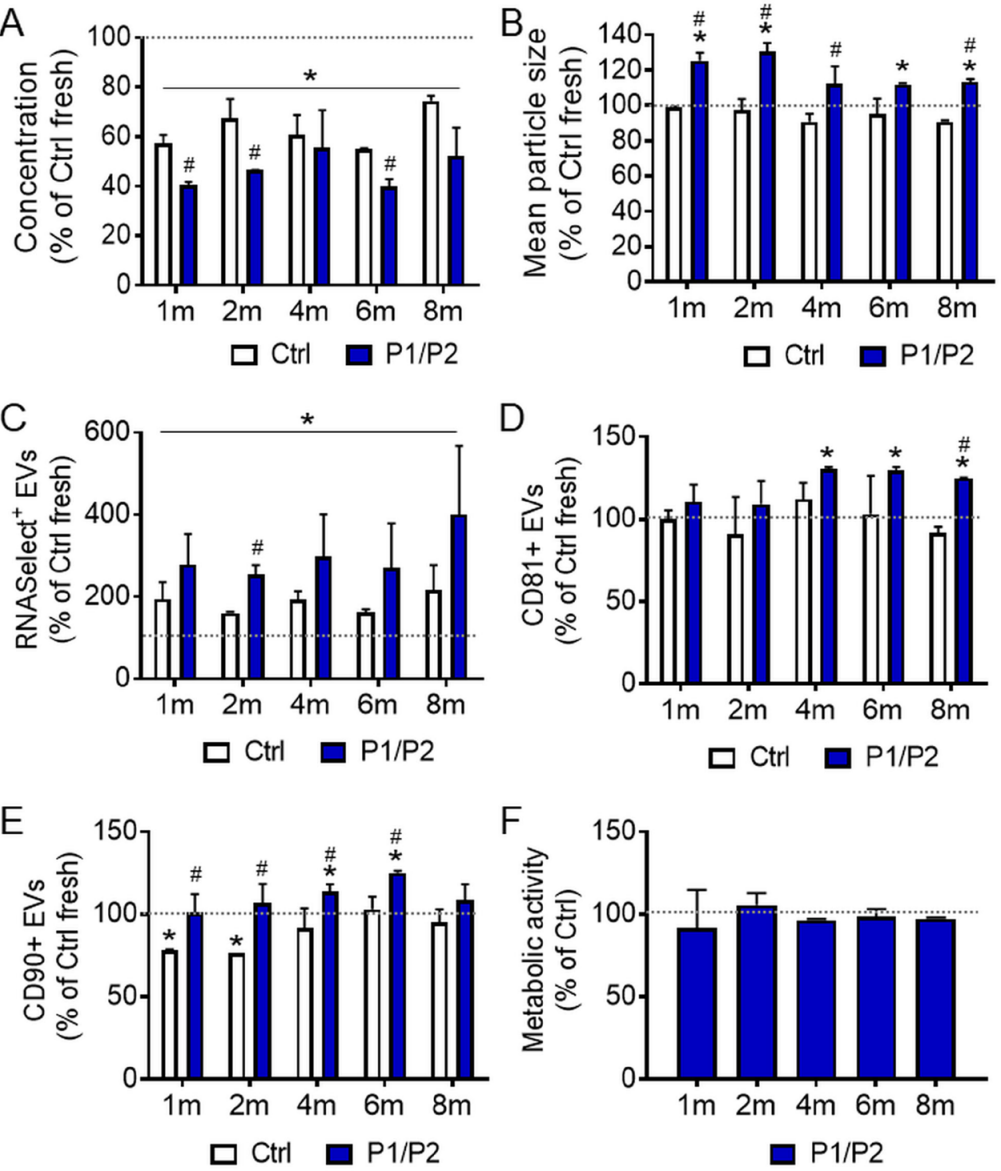
Effect of the long-term cryopreservation of EVs in the presence of P1/P2. Prior analysis, EVs were coated with P1/P2 and cryopreserved for up to 8 months. In subsequent time points, EV samples were thawed and analyzed. NTA analysis of particle concentration (A) and mean size (B) in EV samples after thawing. C-E: Flow cytometry analysis of the cryoprotectant influence the integrity of EVs, assessed by RNASelect dye staining (C), as well as the presence of antigens typical for EVs (CD81; D) and mesenchymal cells (CD90, E). Data on graphs (mean ± SD; *N* = 3) are presented as % relative to Ctrl fresh sample (uncoated EVs, no freezing), indicated by the gray dotted line (100%). (F) Influence of cryopreserved EVs on the metabolic activity of HOBs, measured by the ATPLite kit 4 h after the addition of EVs. Data (mean ± SD; *N* = 3) are presented as % relative to Ctrl samples (uncoated EVs), indicated by the gray dotted line (100%). *P < 0.05 versus Ctrl fresh (uncoated EVs, no freezing). ^#^*P* < 0.05 vs Ctrl (uncoated) EVs in the parallel time point.

We observed significantly decreased EV concentration in both uncoated and P1/P2-coated EV samples in all time points, when compared to the fresh sample (Figure 8A). Additionally, EV concentration was significantly lower in samples coated with P1/P2, compared to uncoated ones in the majority of time points (Figure 8A). However, a similar difference was also seen for EV samples stored at −80 °C for a few days (Figure 2A) and did not significantly change in the subsequent months. Analysis of mean particle size revealed the greater size of EVs coated with P1/P2, when compared to both uncoated and freshly isolated EVs. Again, such an increased size was observed for P1/P2-coated EVs that were frozen for a few days (Figure 2B). This observation confirms the stability of EV coated with P1/P2 within the studied time period (Figure 8B). Additionally, freezing of samples resulted in a significantly higher percentage of RNASelect-positive objects, compared to the fresh samples (Figure 8C). Analogically to the previous results, this may indicate that the freezing-thawing procedure might cause the disintegration of non-EV objects (including contaminants), consequently increasing the overall percentage of RNASelect-positive EV particles. Importantly, coating with P1/P2 significantly improved the stability of EVs, as evidenced by the higher percentage of RNASelect-positive objects when compared to the uncoated samples (Figure 8C).

Additionally, for the majority of time points, the percentage of CD81+ and CD90+ EVs was generally higher in samples coated with P1/P2 than in fresh uncoated EVs and, notably higher than in frozen uncoated EVs (Figure 8D,E), which also proves the stabilizing properties of tested polymers on EVs subjected to the long-term cryopreservation. Finally, we observed a similar functional effect of EVs on the metabolic activity of HOBs as exemplary target cells for both uncoated and P1/P2-coated samples (Figure 8F).

## 4 Discussion

Recent years have brought growing interest in EVs as important factors holding promising potential in several biomedical applications, including diagnostics and therapy. The highest attention is being paid to attempts to utilize EVs as possible therapeutic agents, with special focus on SC-EVs as alternatives to the cell-based approaches.

So far, several studies have demonstrated diverse influence of storage conditions on the stability of EV samples.^33^ However, despite significant advances in the field of EV isolation and characterization, there is still an urgent need to develop and validate their storage conditions that would preserve structural and functional properties. Such effective storage methods are essential elements for the possibility of developing clinical-grade EV preparations with batch-to-batch stability.

Typically, EV storage is based on their preservation in a wide range of temperatures, from −196 °C to +4 °C. However, the understanding of the impact of storage conditions of EV preparations is still limited. Nevertheless, several studies have recognized an effect of storage conditions on downstream applications of EVs, suggesting an important influence of preservation conditions on yield and physical as well as functional properties of EVs. Although EV stability varies depending on their origin and temperature used for their storage, several studies have demonstrated unfavorable structural and biological changes of EV samples, most prominently caused by the destructive effects of water crystals formation, changes in osmotic pressure, aggregation, or degradation, resulting in the reduction of their biological activity.^21^ For example, it has been shown that EVs stored in −80 °C are more stable comparing to +4 °C and −20 °C. Nevertheless, gradual decrease in sample quantity and quality was observed in all storage conditions tested, including severe loss of RNA and protein content.^34^ In another study, EVs produced by hUC-MSCs were shown to be more or less stable for about 7 days at 4 °C. However, their structural integrity was destroyed following freezing-thawing cycles, regardless of tested storage temperature, including −20, −70, or −196 °C.^35^ Similarly, the number, morphology, and total RNA content of EVs isolated from cerebrospinal fluid (CSF) of glioblastoma patients were generally unchanged following their storage at room temperature for up to 7 days, whereas their freezing-thawing resulted in the significant EV sample deterioration.^36^ Apart from the storage temperature, it has been shown that the sample collection and preanalytical manipulation, together with the pH of storage buffer and EV isolation method, may also influence EV stability.^37,38^

To reduce these problems, cryoprotectants dedicated for EV protection need to be developed. Such chemical compounds would prevent hydroosmotic disruption of EVs by increasing the viscosity of their aqueous environment, slowing down the process of ice crystals nucleation. So far, only few substances are known to effectively act as cryoprotectants for whole cells, with dimethyl sulfoxide (DMSO) as the most commonly used one despite its toxicity.^39^ Interestingly, several reports demonstrated that polyampholytes, as polymers containing both cationic and anionic groups (such as carboxylated poly-l-lysine), may also be considered for cell cryopreservation.^40,41^ They were shown to exhibit low toxicity and to act by the reduction of osmotic shock during freezing, but so far, their use has not been extensively examined on the EVs.^42^ Nevertheless, several other substances have been tested as potential EV cryoprotectants, including low molecular weight compounds such as glucose, lactose, sucrose, glycerin, cyclodextrin derivatives, propylene glycol, and trehalose.^43^ DMSO as golden standard cryoprotectant used for cell banking has been also investigated.^35,44^ However, as DMSO is toxic for cells, rapid washing of EV suspension immediately after thawing would be necessary, which requires repurification of EVs, which not only extends significantly the time and increases the cost of EV sample preparation but may also result in their destabilization and disruption.^45^ Additionally, in one study, researchers have demonstrated that neither HEPES nor DMSO could protect EV samples from deterioration throughout their cryopreservation.^46^ Other potential cryoprotectants, such as poly(ethylene glycol) (PEG), poly(vinyl alcohol) (PVA), poly(vinylpyrrolidone) (PVP), and gelatin, could be considered as external cryoprotectants. However, one can expect that they may be effective when used at relatively high concentration, as was demonstrated for mesenchymal stem cells.^20,47−49^ Consequently, these compounds will change the properties of the aqueous phase in which the vesicles are suspended and may interrupt the biological activity of EVs, limiting their therapeutic use. From the practical point of view, utilization of only freshly isolated EV batches is highly limited, especially in the context of their biobanking for clinical purposes. Thus, preservation of EVs during their long-term storage still remains an unsolved challenging problem.

Considering the lack of well-defined conditions for EV long-term storage that would preserve their integrity and biological functions, in the current study, we addressed this issue by developing a new method of EV cryopreservation with ultrathin polyelectrolyte bilayers. Taking into account that EVs are surrounded by the cell membrane that is negatively charged,^50^ we have first coated them with an ultrathin layer of positively charged polyelectrolyte, a block copolymer PEG46-*b*-PMAPTAC52 (P1), assuming that electrostatic and hydrophobic interactions may stabilize the EV membrane. Additionally, we postulated that PEG, which is known to undergo hydration, with one molecule attracting up to 6 water molecules,^51^ reorganizes the layer of water molecules surrounding the EVs, disturbing the ice crystals formation in their vicinity, which creates the layer of “nonfreezable” bound water around the vesicles.^52,53^ We have observed that even a single P1 layer is sufficient to stabilize the hUC-MSC-derived EVs. However, to ensure the biocompatibility of the EVs, we additionally introduced the second negatively charged PAMPS18 (P2) layer using the layer-by-layer technique. Next, we used several complementary methods such as NTA, high-resolution flow cytometry, and mass spectrometry-based proteomic analysis in order to determine an effect of EV coating with P1 and P1/P2 polymers.

Our data indicate stabilizing properties of both P1- and P1/P2-coated EVs following their single freezing-thawing cycle. Based on the NTA data, we observed decrease in the particle concentration of coated EV samples following their thawing, which is a common observation reported in several papers.^54^ However, as, in our study, EVs were isolated from CM by the ultracentrifugation method, the presence of coisolated nonvesicular particles, such as protein aggregates, has to be also taken into account. Importantly, RNASelect staining revealed significantly higher percentage of intact vesicles following cryopreservation of polymer-coated EVs, when compared with the uncoated control. Similarly, the expression of surface-exposed proteins, such as CD81 tetraspanin and CD90, was also higher in EV samples following their coating with P1 and P1/P2. Additionally, in contrast to previous reports, we have not observed a significant increase of particle size following freezing-thawing that would suggest EV aggregation explaining of lowered particle concentration.^19,55^ Altogether, these results may indicate that coating with tested polymers stabilizes EVs and facilitates disruption of other nonvesicular coisolates present in the samples.

Importantly, both polymers exhibited no toxicity in vitro in a wide range of their concentrations when added to the HOBs culture as exemplary mammalian cells. These findings are beneficial when compared to other potential cryoprotectants such as DMSO, which possesses severe cytotoxic effects even at a low concentration.^56^ On the other hand, P1 or P1/P2 coating prior to cryopreservation had a beneficial influence on the functional properties of hUC-MSC-derived EVs. Proregenerative capability of EVs secreted by hUC-MSCs has been previously demonstrated in several studies, both in vitro and in vivo.^57^ In the current study, both tested polymers significantly improved the positive effect of these EVs on the metabolic activity of HOBs, with P1/P2 having a slight positive effect also on the proliferation of these target cells, following their treatment with cryopreserved EVs. Additionally, for both polymers, we demonstrated improved cytoprotective activity when compared to the control samples. As functional activity is essential in the context of practical utility of EVs as therapeutic agents, such results seem to be particularly important for the further development of standardized protocols of EV cryopreservation for their use in clinical practice.

As in the subsequent studies we have focused on the effect of P1/P2 coating, we have also confirmed that P1/P2-coated EVs are structurally stable and do not undergo disintegration even after 20 freezing-thawing cycles, exhibiting no significant changes in their average size but possessing an increased percentage of RNASelect-positive particles, when compared to the uncoated EVs. Similarly, in the long-term storage experiments, when EV samples were cryopreserved in −80 °C for up to 8 months following their coating with P1/P2, we have revealed a similar trend, with decreased particle concentration conjoined with an increased fraction of RNASelect-positive as well as CD81+ and CD90+ particles, when compared to the uncoated EVs. That again suggests the disruption of nonvesicular contaminants, with simultaneous preservation of structural integrity of EVs.

In the next step, we also performed proteomic analysis to evaluate an effect of P1/P2 on EV cryopreservation efficacy. Evaluation of protein content in supernatants and pellets, considered as destroyed vesicles and intact ones, respectively,^19^ confirmed that cryopreservation of EV samples following their coating with P1/P2 allows to protect their protein cargo and structural integrity. In contrast to uncoated control EVs, we demonstrated significantly higher number of identified proteins in P1/P2 ^pellets^, when compared to P1/P2 ^sup^, indicating enrichment in intact EVs in samples coated with P1/P2 prior their freezing-thawing. Additionally, not only the quantity but also quality of proteins in pellets was higher for P1/P2-coated EV samples, showing enrichment in EV- and MSC-related markers.

## 5 Conclusions

This paper presents the novel method for effective and safe cryoprotection of hUC-MSC-derived EVs, based on their coating with ultrathin layers of biocompatible polyelectrolytes using the layer-by-layer technique. Applying the complementary quantitative and qualitative methods, including NTA, high-resolution flow cytometry, proteomics, as well as functional assays in vitro, we have demonstrated that coating of EVs with P1 layer or P1/P2 bilayer protected their structural integrity and biological functions while deep freezing and thawing. The proteomic analysis confirmed that coating of EVs with the P1/P2 bilayer prior their freezing-thawing protects their protein cargo. We believe that our approach constitutes an important step toward the development of a standardized procedure of the long-term storage of EVs for the purpose of basic research as well as clinical applications.

## Supporting Information Available

Additional experimental details, materials, and methods, including photographs of experimental setup, schemes of polymer structures, confocal microscope images, NTA and flow cytometry data, and Venn diagrams of proteomic analysis (PDF)
