# Supplementary material for: Polyelectrolytes Are Effective Cryoprotectants for Extracellular Vesicles
Source: ACS Appl Mater Interfaces. 2024 Dec 12;16(51):70174–86. doi: 10.1021/acsami.4c11852 (PMC11672232; doi:10.1021/acsami.4c11852)
Supplement: Supplementary file 1 — am4c11852_si_001.pdf [file am4c11852_si_001.pdf]

Supporting Information

Polyelectrolytes are effective cryoprotectants for extracellular vesicles

Elżbieta Karnas[a], Mateusz Zając[b], Katarzyna Kmiotek-Wasylewska[a], Kamil Kamiński[b], Shin-Ichi Yusa[c], Sylwia Kędracka-Krok[d], Patrycja Dudek[a], Krzysztof Szczubiałka[b], Maria Nowakowska[b,], Ewa K. Zuba-Surma[a,*]*

[a] Department of Cell Biology, Faculty of Biochemistry, Biophysics and Biotechnology, Jagiellonian University, Krakow 30-387, Poland

[b] Department of Physical Chemistry, Faculty of Chemistry, Jagiellonian University, Krakow 30-387, Poland

[c] Department of Applied Chemistry, Graduate School of Engineering, University of Hyogo, Himeji, Hyogo 671-2280, Japan

[d] Department of Physical Biochemistry, Faculty of Biochemistry, Biophysics and Biotechnology, Jagiellonian University, Krakow 30-387, Poland

*E-mail: nowakows@chemia.uj.edu.pl

*E-mail: ewa.zuba-surma@uj.edu.pl

Supplementary Methods

Reagents

(3-(Methacryloylamino)propyl)trimethylammonium chloride (MAPTAC, 50 wt% in water, Merck, Darmstadt, Germany) was passed through an inhibitor removal column. 2-acrylamido-2-methyl-1-propanesulfonic acid (AMPS, 99%, Merck, Darmstadt, Germany), 4,4′-azobis(4-cyanovaleric acid) (ACVA), 4,4′-azobis(4-cyanopentanoic acid) (V-501, 98%, Sigma-Aldrich, Saint Louis, MO, USA), 4-cyano-4-(phenylcarbonothioylthio)pentanoic acid (CPD) Alexa Fluor 488 C5 maleimide (Molecular Probes, Eugene, OR, US), and methanol (>99%, Merck, Darmstadt, Germany) were used as received. Deionized water was obtained using a Millipore Milli-Q System (Merck).

Apparatus

^{1}H NMR spectra were recorded on an Avance III 600 MHz spectrometer (Bruker, Billerica, MA, US) in deuterated solvents. Gel permeation chromatography (GPC) analysis was performed using a OMNISEC CHR7100 chromatograph (Malvern Pananalytical, Malvern, UK) at room temperature with a right angle light scattering (RALS) detector. The zeta potential of EVs was measured using a Malvern Nano ZS light-scattering apparatus (Malvern Instrument Ltd, Worcestershire, UK). The measurements were carried out at room temperature using the diffusion barrier method according to the manufacturer's protocol. 100 μl of EVs suspension was carefully added to the disposable cuvette filled with 0.7 ml of 0.22 μm-filtered PBS solution. NTA NanoSight NS300 analyzer (Malvern Pananalytical, Malvern, UK) was used to determine the EV concentration and size distribution. Via Freeze controlled rate freezer (Asymptote, Cambridge, England) was used for freezing of EVs at controlled rate. A1-

Si Nikon (Nikon, Tokyo, Japan) confocal laser scanning system built onto a Nikon inverted microscope Ti-E using a Plan Apo 100×/1.4 Oil DIC objective was used to record the EVs images.

Synthesis of PEG46-*b*-PMAPTAC52 (P1)

Cyanopentanoic acid dithiobenzoate (CPD) which was used as a chain transfer agent (CTA), was prepared according to the procedure described in the literature[1]. PEG46-CTA macromonomer, i.e. poly(ethylene glycol) (PEG) with a degree of polymerization equal to 46, terminated with a chain transfer agent (CTA), was prepared according to the procedure described in the publication[1]. MAPTAC (5.00 g, 22.7 mmol), 4,4'-azobis (4-cyanopentanoic acid (V-501), 52.8 mg, 0.188 mmol) and PEG46-CTA (0.887 g, 0.378 mmol) were dissolved in water (37.2 mL). The solution was deoxygenated by purging argon through it for 1 hour. Polymerization was carried out at 70 °C for 20 h. The reaction mixture was dialyzed against pure water for two days. The obtained polymer (PEG46-*b*-PMAPTAC52) was isolated by freeze-drying. 5.03 g of product was obtained (85.4% yield). The structure of the polymer obtained is shown in **Fig. S1.** The polymer has been characterized using spectroscopic and chromatographic methods. The GPC chromatogram of the obtained polymer was measured at 40 °C using an aqueous 0.3 M Na_2SO_4 solution containing 0.5 M acetic acid as an eluent. The number average molecular weight of the polymer, M_n, was 13.8 kDa. The polymer was characterized by a low dispersion coefficient M_w/M_n = 1.16. The number average degree of polymerization (DP) of the PMAPTAC block, determined by ^{1}H-NMR measurements, was 52.

Synthesis of fluorescently labeled PEG46-*b*-PMAPTAC52

PEG46-*b*-PMAPTAC52 was labeled with Alexa Fluor 488. The PEG46-*b*-PMAPTAC52 polymer (200 mg, 0.0146 mmol, was dissolved in methanol (4 mL). $NaBH_4$ (55.2 mg, 1.46 mmol) was added to the solution and stirred for 5 hours. Then, the reacted solution was dialyzed against pure water for 4 h. After dialysis, Alexa Fluor 488 C5-maleimide (1 mg dissolved in 1 mL of water) was added to the solution and stirred for 24 h. After the reaction, the solution was dialyzed against pure water for 3 days. Labeled PEG46-*b*-PMAPTAC52-AF488 was recovered using a freeze-drying technique (137 mg, 68.4%). The modified polymer was characterized by GPC, NMR, FTIR and elemental analysis. The content of Alexa Fluor 488 in the polymer accounted for 1.79 mol%.

Synthesis of PAMPS18 homopolymer (P2)

PAMPS18 homopolymer (where *x* denotes the degree of polymerization, DP) was obtained by the RAFT method (**Fig. S2**). Briefly, AMPS (2-acrylamido-2-methylpropanesulfonic acid) (5.00 g, 24.1 mmol) was neutralized with 6 M NaOH (5.8 mL) to a pH of 6.03. 4,4′-azobis(4-cyanopentanoic acid) (V-501, 18.0 mg, 0.0643 mmol) and 4-cyano-4-(phenylcarbonothioylthio)pentanoic acid (CPD, 44.9 mg, 0.161 mmol) were dissolved in a mixture of 2 mL of MeOH and 16 mL of water, and then added to the aqueous AMPS solution. The solution was degassed by purging argon through it for 30 min. Polymerization was carried out at 70 °C for 2 h. The reaction mixture was dialyzed against pure water for one day. PAMPS142 was isolated by freeze-drying (4.88 g, 96.7%). The number average molecular weight (M_n(NMR)), the degree of polymerization (DP) determined by ^{1}H NMR and the molecular weight dispersity coefficient (M_w/M_n) determined by gel permeation chromatography (GPC) were 4.0 kDa, 18 and 1.21, respectively.

Cell culture

Human umbilical cord mesenchymal stem cells (hUC-MSCs) were isolated with explants method according to the previously described protocol[2] and expanded in DMEM/F12 (Sigma-Aldrich, Saint Louis, MO, USA) medium containing 10% addition of fetal bovine serum (FBS; Sigma-Aldrich), penicillin and streptomycin (100 U/ml; Thermo Fisher Scientific, Waltham, MA, USA) in standard conditions (5% CO_2, 37 ^{o}C). Primary human osteoblasts (HOBs; PromoCell, Heidelberg, Germany) were cultured in standard conditions (5% CO_2, 37 ^{o}C) in dedicated Osteoblast Growth Medium (OGM; PromoCell) supplemented with penicillin and streptomycin.

Isolation of EVs

EVs were isolated from the conditioned medium (CM) harvested from hUC-MSCs, using the sequential centrifugation method (Fig. S3). Prior to CM collection, hUC-MSCs were seeded and cultured in ultracentrifuged media, in order to eliminate EVs and small particles of FBS origin. Media were ultracentrifuged (100 000 g, 18 h, 4 ^{o}C) using Optima XPN-90 ultracentrifuge and type 50.2 Ti fixed-angle rotor (Beckman Coulter, Brea, CA, USA). Collected media supernatants were then used for the cell culture.

For EV isolation, after the preliminary CM centrifugation (2000 g, 20 min, 4 °C) to get rid of cellular elements and larger cell debris, CM supernatants were ultracentrifuged at 100 000 g for 70 min at 4 °C to pellet the EV fraction. Pellets were rinsed with 0.22 µm-filtered phosphate buffered saline devoid of calcium and magnesium ions (PBS; Lonza, Basel, Switzerland), and then ultracentrifuged again with the same parameters. Obtained pellets containing EVs were resuspended in PBS.

Coating with one layer of polymeric cryoprotectant

EVs were coated with the polymer immediately after the isolation. For that purpose EVs were suspended in 200 µl of PBS solution. The suspension was then divided into two parts of equal volumes. One part (100 µl) was mixed at room temperature (RT) with P1 (1 mg/ml) solution in PBS (5.3 µl). In the resulting mixture, the concentration of P1 was 50 µg/ml and the concentration of EVs was 10^{11}/ml. An equal volume of PBS solution (5.3 µl) was added to the second part of EV suspension and was used as a control (Ctrl) sample.

Coating with two layers of polymeric cryoprotectants

EVs previously coated with P1 were additionally coated with P2. 1 mg/ml P2 solution (in PBS) was added to EVs to the final concentration of 50 µg/ml. Thus, the weight ratio of the P1 and P2 polymers was 1:1. Sample containing EVs coated with a bilayer of polymers (PEG_{46}-*b*-$PMAPTAC_{52}$ and $PAMPS_{18}$) is further referred to as P1/P2.

Cryopreservation of EVs

Control EVs (Ctrl) or EVs coated with one (P1) or two (P1/P2) polymeric layers were frozen in -80°C at the controlled rate of -1 °C/min and stored at -80 ^{o}C for minimum five days. Subsequently, samples were thawed by heating in a 37 °C water bath until ice crystals disappeared and were subjected to the further analyses. EV samples were either frozen and thawed one time (single freezing-thawing cycle) or multiple times (10x or 20x) prior to analyses. Additionally, long-term storage (up to 8 months) approach was also performed, where samples were frozen and then thawed one time after 1, 2, 4, 6 and 8 months of storage in -80°C.

Confocal microscopy

Visualization of EVs polymeric coating was done with scanning confocal microscopy (laser excitation at 488 nm, FITC channel) using a cationic block copolymer labeled with a Alexa Fluor 488 molecular fluorescent marker (PEG46-PMAPTAC52-AF488).

Nanoparticle tracking analysis (NTA)

In order to evaluate the concentration and size distribution of EVs, NanoSight NS300 analyzer (Malvern Pananalytical, Malvern, UK) was used. For the measurement, EV samples were 1000x diluted in 0.2 μm- filtered DPBS into the final volume of 3 ml. Each sample tracking was performed with three repetitions, with camera level 13 and threshold parameter set on 2. Mean and mode size of particles as well as their concentration were calculated using NTA Software ver. 3.4 (Malvern Pananalytical).

High-resolution flow cytometry

EVs were stained with buffer containing SYTO RNASelect dye (Thermo Fisher Scientific) and one of allophycocyanin (APC)-conjugated mouse monoclonal antibodies: anti-anti-CD81 (clone 5A6), CD90 (clone 5E10), or appropriate isotype-match control (all from BioLegend, San Diego, CA, USA). Prior to staining, RNASelect dye (at final concentration of 5 μM) and appropriate antibodies were resuspended in 0.2 μm-filtered DPBS and centrifuged at 21 000 g for 20 min at 4 ^{o}C to remove potential debris and protein aggregates. Next, supernatants of staining buffers were transferred into fresh tubes and EV samples were added for 30 minutes in 4 ^{o}C. Flow cytometry analysis was performed with Apogee A60-Micro-PLUS cytometer and Histogram software (Apogee Flow Systems, Hemel Hempstead, UK). Prior to analysis, the system was calibrated using dedicated Apogee Calibrating Beads (#1493; Apogee Flow

Systems). Additionally, samples containing only staining buffer were also acquired to verify the absence of potential background particles.

Cytotoxicity assay *in vitro*

In order to test an effect of P1 and P1/P2 on the mammalian cells, HOB cells were seeded onto black 96-well plate (Corning, Corning, NY, USA) in the number of 2×10^3 of cell per well. After 24 h, polymers were added for 24 h into the culture medium at the concentration of 5, 50 and 500 µg/ml. Cells untreated with polymers served as a control. Subsequently, proliferation, cytotoxicity and apoptosis of HOBs were assessed by ApoTox-Glo Triplex Assay kit (Promega, Madison, WI, US), according to the manufacturer’s protocol. Fluorescent and luminescent signals from individual wells were acquired using Infinite M200 Pro analyzer and i-control Software (Tecan, Männedorf, Switzerland).

Viability assay

Prior to the treatment with EVs, HOB cells were seeded onto 96-well black plate (Corning) in the number of 2×10^3 of cells per well. After 24h, previously cryopreserved uncoated EVs (Ctrl) or EVs coated with P1 or P1/P2 were added into wells in the dose of 1×10^9 particles per well. Cells treated with non-cryopreserved uncoated EVs (Ctrl fresh) served as a control. After 48 h cell viability was measured using CaspGlo Luminescence Assay (Promega), according to the manufacturer’s protocol. Luminescent signals from individual wells were acquired using Infinite M200 Pro analyzer and i-control Software (Tecan).

Metabolic activity assay

Prior to treatment with EVs, HOB cells were seeded onto 96-well white plate (Perkin Elmer, Waltham, MA, USA) in the number of 2×10^3 of cells per well. After 24h, previously cryopreserved uncoated EVs (Ctrl) or EV coated with P1 or P1/P2 were added into wells in the dose of 1×10^9 particles per well. Cells treated with non-cryopreserved uncoated EVs (Ctrl fresh) served as a control. Cells were incubated in standard conditions for 4 h. After that time the concentration of ATP produced by the cells was measured by ATPLite Lumiescence Assay System (Perkin Elmer), according to the manufacturer’s protocol. Luminescent signals from individual wells were acquired using Infinite M200 Pro analyzer and i-control Software (Tecan).

Cytoprotection assay

HOB cells were seeded onto 96-well black plate (Corning) in the number of 2×10^3 of cells per well. After 24 h, cells were treated with staurosporine as an inducer of apoptosis (1 μM; Sigma Aldrich) for 4 h. Next, previously cryopreserved uncoated EVs (Ctrl) or EVs coated with P1 or P1/P2 were added into wells in the dose of 1×10^9 particles per well. Cells treated with non-cryopreserved uncoated EVs (Ctrl fresh) served as a control. After 4 h cell viability was measured using CaspGlo Luminescence Assay (Promega), according to the manufacturer’s protocol. Luminescent signals from individual wells were acquired using Infinite M200 Pro analyzer and i-control Software (Tecan).

Proteomic analysis

EVs coated with P1/P2 were subjected to a single freezing-thawing cycle. Prior to coating, EV samples were additionally purified from protein aggregate co-isolates

using Izon qEV single 70 nm columns (Izon Science, Lyon, France), according to the manufacturer’s protocol. Control (uncoated) or P1/P2-coated EVs were divided into two equal volumes of non-cryopreserved (fresh) and cryopreserved samples. Following single freezing-thawing cycle, cryopreserved samples were ultracentrifuged (100 000 g, 70 min at 4 °C) to separate pellets containing intact EVs from supernatants containing proteins released by the potential disruption of EVs. Pellet and supernatant samples, as well as fresh control samples were subjected to proteomic analyses.

Sample preparation for LC-MS/MS analysis

SDS in 50 mM triethylammonium bicarbonate (TEAB; pH 7.55) was added to the samples to the final concentration of 5 %. The samples were then sonicated for 15 min at 320 W (intensity setting – high) with a 30 s/30 s ON/OFF time interval using a Bioruptor UCD-200 sonicator (Diagenode, Liege, Belgium). The samples were then incubated at 95 °C for 5 min and centrifuged (20,000×g, 10 min, RT). Supernatants were preserved for further analysis. The protein concentration of the samples was measured using a tryptophan fluorescence assay[3]. For LC-MS/MS analysis 25 □g of each sample was taken. Protein digestion was performed using S-Trap™ micro spin columns according to the manufacturer’s protocol. Briefly, samples were reduced with 50 mM DTT solution, and alkylated with the addition of iodoacetamide to a final concentration of 40 mM, then acidified with phosphoric acid. Afterwards, 6 volumes of Strap binding buffer (90% aqueous methanol, 100 mM TEAB, pH 7.1) were added and the mixture was placed on the S-Trap by centrifugation at 4000× g for 10 s. Samples were purified by washing with S-Trap binding buffer and centrifugation. Proteins were digested overnight with trypsin (Promega, Madison, WI, USA) at 20:1 protein-to-enzyme wt:wt ratio. Peptides were eluted with 50 mM TEAB, followed by 0.2% v/v

aqueous formic acid (FA) and 50% v/v acetonitrile, containing 0.2% v/v FA. Peptide samples were desalted using C18 Empore™ Solid Phase Extraction (SPE). Lastly, peptides were vacuum dried.

Liquid chromatography and tandem mass spectrometry (LC-MS/MS) measurement

Peptides were analyzed using Q Exactive high-resolution mass spectrometer (Thermo Fisher Scientific) with DPV-550 Digital PicoView nanospray source coupled with nanoHPLC an UltiMate 3000RS LC nanoSystem (Dionex) Peptides were loaded on a C18 precolumn (Acclaim PepMap Nano trap Column) using 2% v/v acetonitrile with 0.05% v/v TFA as a mobile phase, and further separated on a 50 cm×75 μm RP column (Acclaim PepMap 75 μm 100 Å Nano Series TM Column) with gradient 2–40% ACN in 0.05% FA for 240 minutes. Full MS scans were acquired in the Orbitrap mass analyzer over m/z 300–2000 range with resolution 70,000 (at m/z 200). The top twelve most intense peaks with charge state ≥ 2 were fragmented in the HCD collision cell normalized collision energy of 27%, (the isolation window was 1.2 m/z). Tandem mass spectrum was acquired in the Orbitrap mass analyzer with resolution 17,500 at m/z 200.

LC-MS/MS data analysis

The RAW LC-MS/MS files were analyzed using MaxQuant 1.6.7.0 and an Andromeda server against the SwissProt database with Homo sapiens taxonomy restriction (20385 sequences) supplemented with common protein contaminant database. iBAQ (Intensity Based Absolute Quantification, i.e., Σ intensity/#theoretical peptides) quantification was enabled and standard software settings were used, which included

a false discovery rate (FDR) below 1% for peptide and protein identification. The search parameters were as follows: enzyme - trypsin; number of missed cleavages - 2; static modification – carbamidomethylation (C); dynamic modifications - oxidation (M) and Acetyl (Protein N-term). Statistical analysis was done using Perseus software 1.6.7.0. Protein groups from the reverse database, common protein contaminants, as well as proteins only identified by site, were filtered out (with 3909 protein groups left).

Supplementary Figures

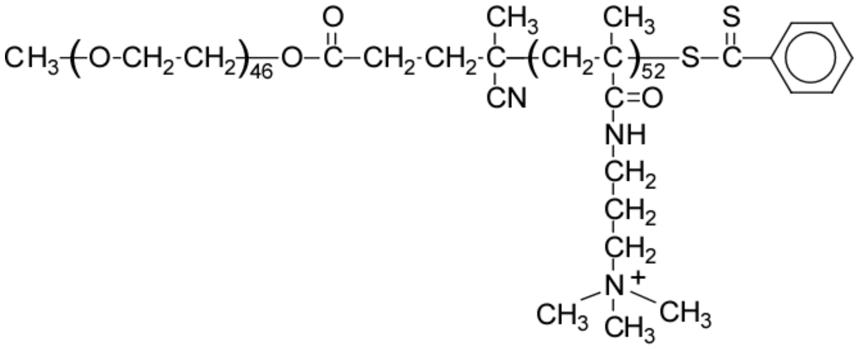

Figure S1. Structure of PEG46-*b*-PMAPTAC52 copolymer.

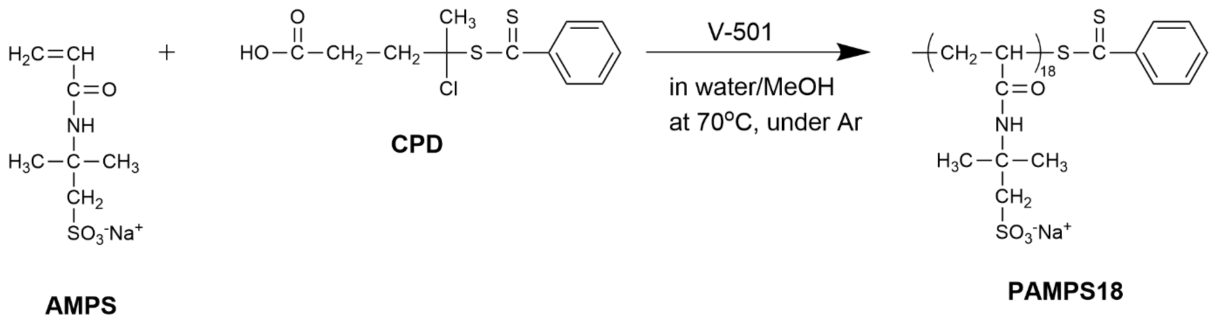

Figure S2. Synthesis of PAMPS18 homopolymer.

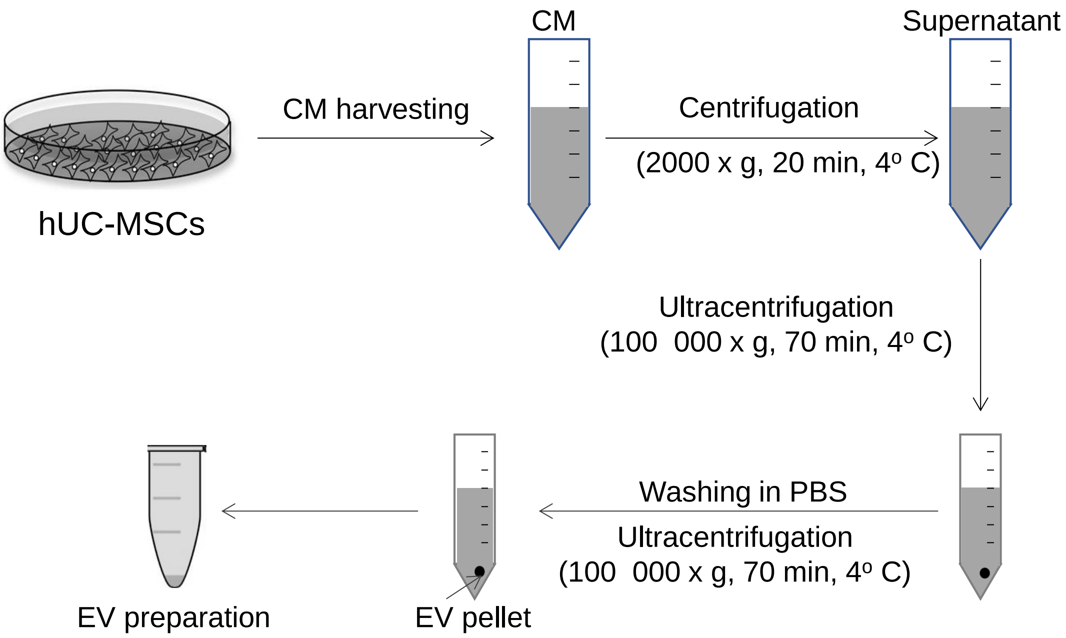

Figure S3. Schematic diagram of the EVs isolation procedure involving sequential centrifugation with an ultracentrifugation step. CM – conditioned medium.

A

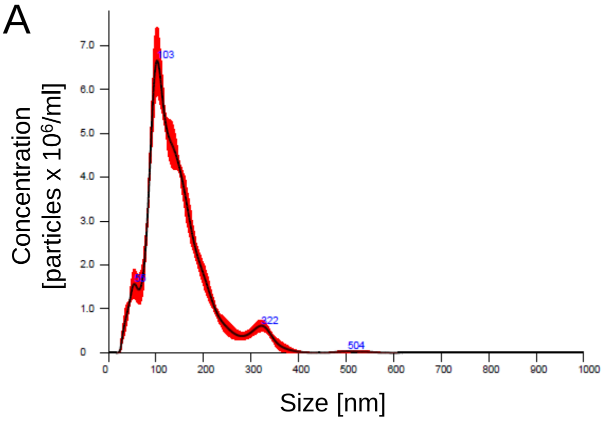

B

Parameter		[nm]
Diameter	Mean	150.2 ± 6.7
	Mode	117.3 ± 6.9

Figure S4. NTA analysis of EVs derived from hUC-MSCs. A: Size distribution of particles in freshly isolated EV samples. Representative histogram of size distribution was shown. **B**: Cumulative data obtained from experimental repetitions, presented as the mean and mode ± SD (N=3).

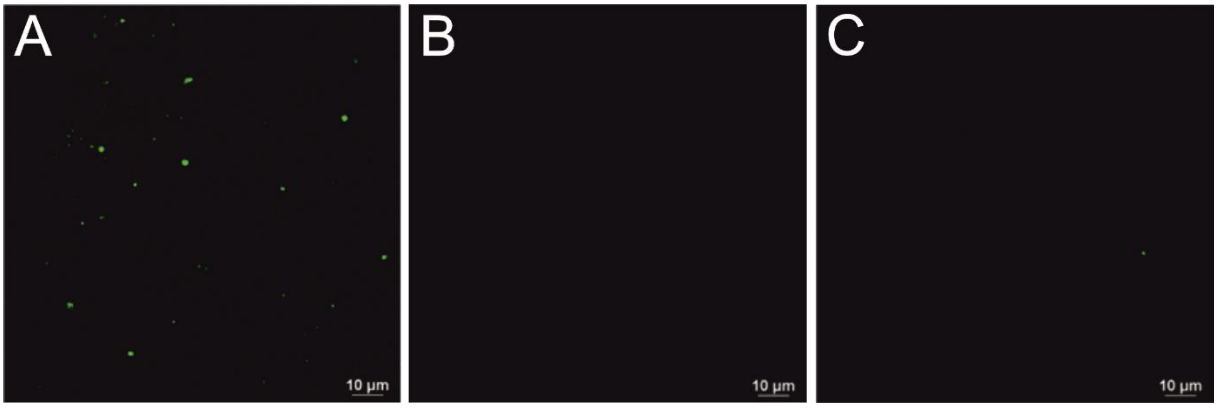

Figure S5. EVs imaging using a confocal microscope. A) EVs coated with PEG46-*b*-PMAPTAC52-AF488 polymer, B) EVs without polymer addition (control), C) PEG46-*b*-PMAPTAC52-AF488 polymer solution.

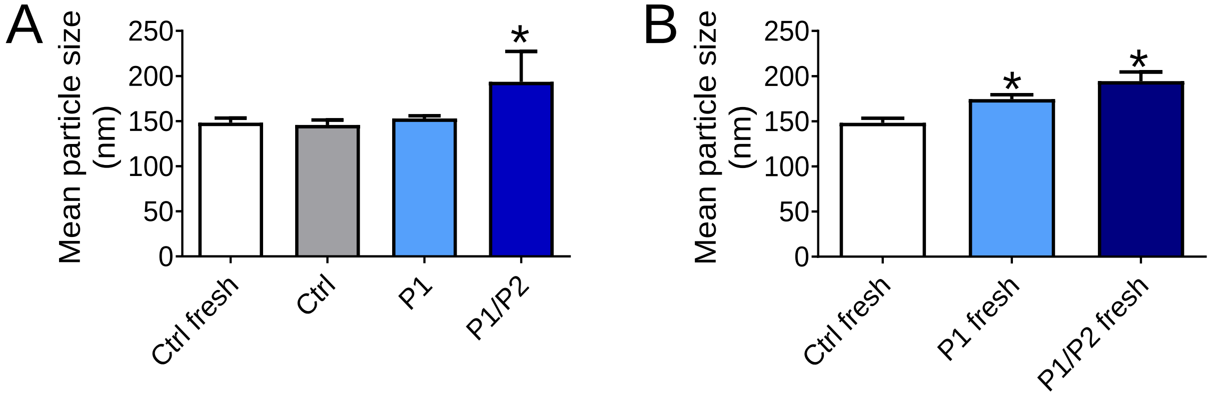

Figure S6. An effect of single freezing-thawing cycle on the mean size of particles in EV samples in the presence of polymeric cryoprotectants P1 and P1/P2. NTA analysis of mean size expressed in nm for freshly isolated uncoated EV samples vs. samples after freezing-thawing process (A) or vs. coated EV samples prior to their freezing-thawing (B).

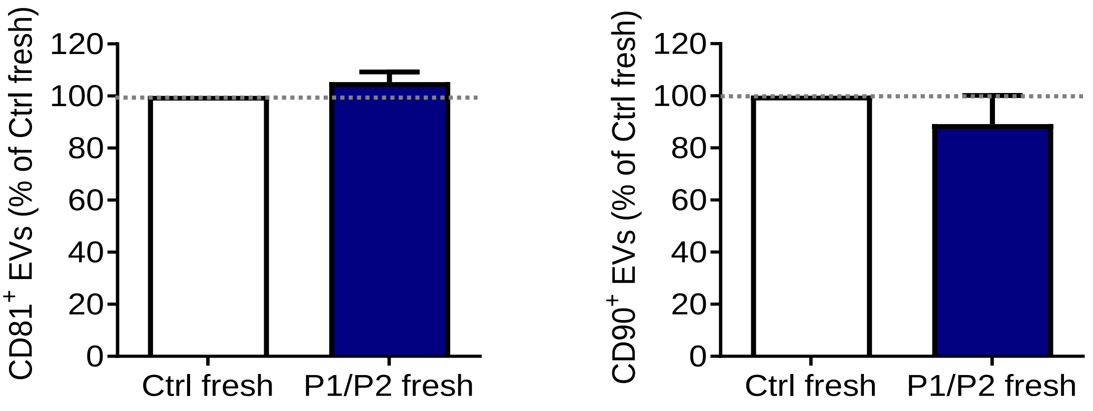

Figure S7. An effect of EV coating with P1/P2 bilayer on the binding of fluorochrome-conjugated antibodies against CD81 and CD90 antigens. Flow cytometry data (mean ± SD; N=3) for freshly isolated uncoated (Ctrl fresh, 100%) or P1/P2 bilayer-coated EV samples.

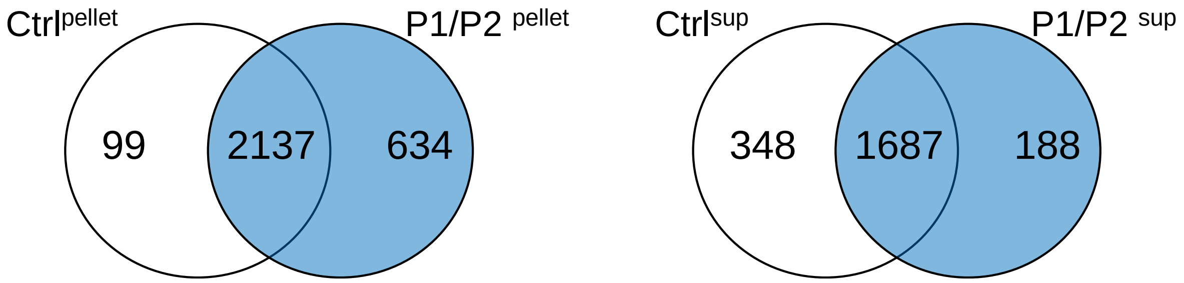

Figure S8. Proteomic analysis of EVs cryopreserved following coating with P1/P2. Venn diagrams of analysis performed for pellets (left) and supernatants (right) comparing the number of proteins identified among all proteins detected in minimum 2 sample repetitions. Middle value corresponds to the number of common proteins (present in both sample types), whereas extreme values correspond to the unique proteins (present exclusively in one sample type among two compared groups).

Supplementary Tables

Table S1. The values of zeta potential of uncoated (Ctrl) EVs and EVs coated with P1 layer or (P1/P2) bilayer before and after one freezing-thawing cycle

Sample	Zeta potential [mV]		
	**EVs Ctrl**	**EVs coated with P1**	**EVs coated with P1/P2**
**Before freezing**	-17.3±0.3	-8.2± 0.4	-13.7±0.9
**After one cycle of freezing-thawing**	-19.0±0.9	-8.4±0.2	-14.4±1.3

Supplementary References

(1) Mitsukami, Y.; Donovan, M. S.; Lowe, A. B.; McCormick, C. L. Water-Soluble Polymers. 81. Direct Synthesis of Hydrophilic Styrenic-Based Homopolymers and Block Copolymers in Aqueous Solution via RAFT. *Macromolecules* **2001**, *34* (7), 2248–2256. DOI: 10.1021/ma0018087.

(2) Bobis-Wozowicz, S.; Kmiotek, K.; Kania, K.; Karnas, E.; Labedz-Maslowska, A.; Sekula, M.; Kedracka-Krok, S.; Kolcz, J.; Boruczkowski, D.; Madeja, Z.; Madeja, Z.; Zuba-Surma, E. K. Diverse Impact of Xeno-Free Conditions on Biological and Regenerative Properties of HUC-MSCs and Their Extracellular Vesicles. *J. Mol. Med.* **2017**, *95* (2), 205–220. DOI: 10.1007/s00109-016-1471-7.

(3) Wiśniewski, J. R.; Gaugaz, F. Z. Fast and Sensitive Total Protein and Peptide Assays for Proteomic Analysis. *Anal. Chem.* **2015**, *87* (8), 4110–4116. DOI: 10.1021/ac504689z.
